# Single-nucleus RNA sequencing: immature excitatory neurons and transformed glia build human BRAF^V600E^-negative gangliogliomas

**DOI:** 10.1093/braincomms/fcaf372

**Published:** 2025-10-01

**Authors:** Silvia Cases-Cunillera, Philipp Müller, Ashley J van Waardenberg, Susanne Schoch, Gilles Huberfeld, Anke Höllig, Daniel Delev, Motaz Hamed, Albert J Becker, Karen M J van Loo

**Affiliations:** Université Paris Cité, Institute of Psychiatry and Neuroscience of Paris (IPNP), ISERM U1266, Neuronal Signaling in Epilepsy and Glioma, Paris 75014, France; Institute for Cellular Neurosciences II, Medical Faculty, University of Bonn, Bonn 53127, Germany; i-Synapse, Cairns 4870, Australia; Institute for Cellular Neurosciences II, Medical Faculty, University of Bonn, Bonn 53127, Germany; Université Paris Cité, Institute of Psychiatry and Neuroscience of Paris (IPNP), ISERM U1266, Neuronal Signaling in Epilepsy and Glioma, Paris 75014, France; Department of Neurology, Hopital Fondation Adolphe de Rothschild, Paris 75019, France; Department of Neurosurgery, RWTH Aachen University Hospital, Aachen 52074, Germany; Department of Neurosurgery, RWTH Aachen University Hospital, Aachen 52074, Germany; Department of Neurosurgery, Universitätsklinikum Erlangen, Friedrich-Alexander-Universität Erlangen-Nürnberg, Erlangen 91054, Germany; Department of Neurosurgery, University Hospital Bonn, Bonn 53127, Germany; Institute for Cellular Neurosciences II, Medical Faculty, University of Bonn, Bonn 53127, Germany; Department of Neurosurgery, RWTH Aachen University Hospital, Aachen 52074, Germany; Department of Epileptology, Neurology, RWTH Aachen University Hospital, Aachen 52074, Germany

**Keywords:** neuronal development, glutamatergic, immature excitatory neurons

## Abstract

Gangliogliomas are glioneuronal neoplasms, generally with a benign evolution, accounting for the most common tumours in patients with long-term pharmacoresistant epilepsy. *BRAF^V^*^600*E*^ has been detected in a high percentage of World Health Organization grade 1 gangliogliomas. However, biopsy collections from epilepsy patients include tumours that meet neuropathological ganglioglioma criteria but lack the *BRAF^V^*^600*E*^ mutation. The molecular pathology of such BRAF^V600E^-negative gangliogliomas remains largely unexplored. We here focused on decoding the molecular and cellular profiles of these BRAF^V600E^-negative gangliogliomas at the single-cell resolution. Our results identify an exclusive profile in excitatory, but not inhibitory, neurons, astrocytes and oligodendrocytes in BRAF^V600E^-negative gangliogliomas. Moreover, we confirm the presence of immature excitatory neurons with higher expression of genes related to glutamatergic transmission as well as metabolically active astro- and oligodendrocytes which may contribute to epileptogenicity. Our study provides important resources to understand the molecular profile of BRAF^V600E^-negative gangliogliomas aiming to drive forward future research directions to novel and tailored treatment options for epilepsy.

## Teaser

Cases-Cunillera et al. report that BRAF^V600E^-negative gangliogliomas display immature excitatory neurons with enhanced glutamatergic signaling and metabolically altered glial populations. These findings uncover distinct transcriptional signatures underlying epileptogenicity and highlight potential therapeutic targets beyond the Mitogen-Activated Protein Kinase (MAPK) pathway.

## Graphical Abstract

**Graphical Abstract:**
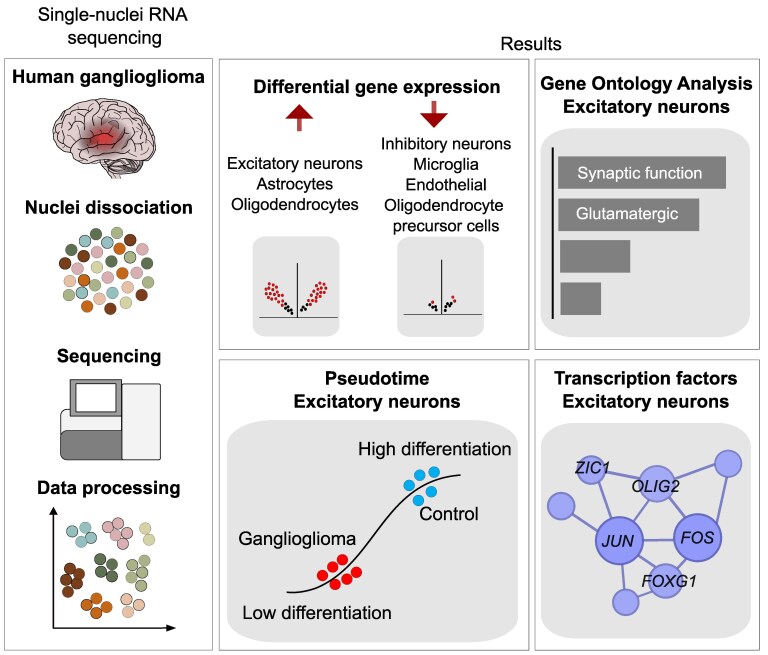

## Introduction

Gangliogliomas (GGs) are glioneuronal neoplasms composed of dysmorphic neurons and neoplastic glial cells, accounting for the most common tumour associated with long-term pharmacoresistant epilepsy.^1^ The course and nature of GGs are generally benign and surgical resection allows seizure control in a large fraction of patients.^2-4^ However, epileptic seizures are a factor of concern since they can remain after surgery.^5^ Therefore, finding new treatments to tackle this symptomatology in patients with GGs is critical.

BRAF^V600E^ mutation has been reported in a considerable fraction of WHO grade 1 GGs ranging from 13% to 58% depending on the study.^6-10^ Regardless of the discrepancy in the percentage of GGs harbouring the mutation, all studies identified BRAF^V600E^-positive GGs within their cohorts. This has resulted in research focusing on understanding molecular mechanisms associated with this mutation in both human and mouse GGs^7,11-14^ and led to BRAF^V600E^-negative tumours that clearly fulfil histological and immunohistochemical criteria of GG being less investigated.^15^ Even though BRAF^V600E^ has been associated with neuronal hyperexcitability^13,16^ previous reports have claimed no significant differences in epilepsy duration^17^ or age at seizure onset^6,18^ between patients with GG with or without BRAF^V600E^ mutation.

In this study, we aim to unravel molecular features of human tumours fulfilling histological and immunohistochemical criteria of GGs but BRAF^V600E^ negative status, further referred to as GGs, from patients with a clinical story of epilepsy at a single-cell resolution.

## Materials and methods

### Human samples

GG (WHO grade 1) samples negative for BRAF^V600E^ were obtained from patients who underwent surgery at the Department of Neurosurgery of the University of Bonn. Control samples were collected at the Department of Neurosurgery of the RWTH Aachen University Hospital. The clinical courses of the patients are summarized in Supplementary Tables 1 and 2. All procedures were conducted in accordance with the Declaration of Helsinki. Informed written consent was obtained from every patient according to the approvals of the local ethics committee (University of Bonn: Nr. 229/18, 371/20, 504/20; RWTH Aachen University Hospital: EK067/20). Relevant clinical information was extracted from the corresponding medical reports.

### Histology

Brain specimens were fixed in paraformaldehyde and embedded in paraffin. The resulting paraffin-embedded blocks were sectioned at a thickness of 4 µm. The paraffin was removed by sequential treatment with ethanol solutions of decreasing concentrations (1 min each). Following deparaffinization, the brain sections were washed with distilled water and immersed in haematoxylin for 5 min. After an additional wash with crane water, the sections were treated with eosin for 2 min. To remove excess eosin, the sections were washed with 100% ethanol for 30 s, followed by two washes with isopropanol (2 min each) and xylene (1 min each). After air-drying for 5 min, a coverslip was mounted on the haematoxylin and eosin (H&E)-stained brain sections using Cytoseal. BRAF^V600E^ status was validated by immunochemistry using the anti-BRAF-V600E (VE1) Mouse Monoclonal Primary Antibody (VENTANA) (Supplementary Fig. 2B).

### Tissue dissociation to single nucleus

A single nuclei suspension was prepared from frozen human brain samples stored at −80°C. The samples were sectioned into small fragments and resuspended in ice-cold lysis buffer (Miltenyi Biotec). The resulting suspension was transferred to a gentleMACS C tube (Miltenyi Biotec) and processed using the gentleMACS Dissociator (Miltenyi Biotec) to achieve tissue dissociation into nuclei. Subsequently, the nuclei suspension was filtered through 70 µm and 30 µm MACS SmartStrainers (Miltenyi Biotec). The filtered solution was centrifuged, the supernatant was removed and the pellet was then resuspended in an ice-cold resuspension buffer by gentle pipetting. The nuclei suspension was centrifuged, and the resulting pellet was resuspended in phosphate-buffered saline (PBS). Debris Removal Solution was added, followed by the careful addition of PBS. After centrifugation at full acceleration, three distinct phases formed; the two upper phases, containing the debris, were aspirated and discarded. The remaining solution was centrifuged, and the pellet was resuspended in an appropriate volume of buffer (PBS containing 2% bovine serum albumin, BSA). The nuclei were treated with DAPI at a final concentration of 0.1 µg/mL. All solutions were supplemented with an RNase inhibitor at a final concentration of 0.5%. The nuclei suspension was maintained in ice for all the steps. Nuclei concentration was determined using a Countess automated cell counter.

### Single-nucleus RNA sequencing

Single-nucleus RNA sequencing (snRNA-seq) was performed using the 10 × Genomics Chromium NextGEM Single Cell 3′ Reagent Kits v3.1, following the manufacturer’s instructions. Nuclei were loaded onto the GEM Chip at a concentration of 500–1500 nuclei/µL, targeting 16 500 nuclei per sample. Libraries were sequenced on a NovaSeq 6000 platform with read settings of 28/10/10/90, achieving an average sequencing depth of ∼25 000 reads per nucleus.

### Single-nucleus RNA sequencing data pre-processing

snRNA-seq data from samples from temporal lobe epilepsy (TLE; referred to as non-GG^ctrl^) and GGs was mapped against the ‘GRCh38-3.0.0’ human reference transcriptome (downloaded from 10xgenomics: https://cf.10xgenomics.com/supp/cell-exp/refdata-cellranger-GRCh38-3.0.0.tar.gz) with Cell Ranger version 6.1.2.^19^ Fastq sequencing data corresponding to each sample were input into the CellRanger ‘count’ subcommand, with parameters –expect set to 10 000 and run two times, firstly with ‘-include-introns’ and secondly without ‘-include-introns’. The snRNA-seq data from^20^ were retrieved from the Gene Expression Omnibus (GEO:GSE168408). All datasets were integrated and analysed using the Seurat package (v4.0). Filtered feature-barcode matrices were loaded into R using the *Read10X_h5* function. Each dataset was converted into a Seurat object using *CreateSeuratObject* function with a minimum feature threshold of 100 genes per nucleus.

Quality control was performed by filtering nuclei based on the following criteria: exclusion of (1) nuclei with fewer than 250 genes or 500 UMIs, (2) nuclei with a mitochondrial gene ratio greater than 20% and (3) nuclei with less than 0.8 log_10_GenesPerUMI ratio. Filtered datasets were merged using *Merge* function to create a combined dataset containing foetal, neonatal, childhood, and adult developmental samples alongside non-GG^ctrl^ and GG samples. The full names of gene symbols which are displayed in Figures and Supplementary figures are listed in Supplementary Table 4.

### Dimensionality reduction and cell type annotation

To correct batch effects, each dataset underwent normalization using the *NormalizeData* function with default settings, ensuring consistency across samples. To identify the most informative genes, the top 2000 variable features were selected using *FindVariableFeatures* function. The datasets were integrated using *IntegrateData*, incorporating the first ten principal components to create a harmonized dataset suitable for downstream analyses. After integration, standard Seurat workflows were applied to reduce dimensionality and identify distinct cell populations. The integrated dataset was first scaled using *ScaleData* function and subjected to *RunPCA* function to capture major sources of variation. To visualize the data in a lower-dimensional space, *RunUMAP* function was performed using the first ten principal components. Clustering was then conducted using a graph-based approach, where nearest-neighbour graphs were computed with *FindNeighbors* function, followed by clustering at a resolution of 0.5 using *FindClusters* function. Cell type annotation was performed based on marker gene expression profiles, leveraging *FeaturePlot* and *VlnPlot* functions to examine established markers.

### Trajectory analysis

To analyse developmental trajectories, the Seurat object was converted into a Monocle3 cell dataset, preserving metadata, gene features, and clustering information. UMAP coordinates were transferred to maintain spatial distribution, and all cells were assigned to a single partition. Trajectory inference was performed using Monocle3’s graph-learning algorithm, reconstructing differentiation pathways and identifying key transition points such as branch points and root cells. To assign the root in Monocle3, we utilized cells from the fetal group of the GEO:GSE168408 dataset, ensuring accurate pseudotime ordering. Cells were then ordered along the trajectory, with pseudotime values assigned to track excitatory neuron differentiation.

### Copy number variation analysis

Analysis was performed with R package inferCNV (version 1.16.0). Raw gene expression counts were extracted from the Seurat object using the RNA assay. Genes with an average expression below 0.1 in the reference group were excluded (cut-off = 0.1). Annotations linking each cell to its cell type were provided as input to *CreateInfercnvObject* function, and gene position information was supplied via a custom file. The CNV inference was performed using *infercnv::run* function with denoising (denoise = TRUE), a six-state Hidden Markov Model.

### Transcription factor enrichment analysis

Enrichment of transcription factors (TFs) was determined using the ChIP-X Enrichment Analysis Version 3 API (ChEA3).^21^ The ten most enriched transcription factors are displayed along with their mean rank. TFEA data were embedded in the STRING functional association network.^22^

### Statistical analysis

Violin plots were generated to visualize gene expression distributions across cell populations identified in the single-cell RNA-seq dataset. Statistical comparisons underlying these plots were performed using Seurat’s *FindAllMarkers* function. Violin plots showing gene expression between different conditions were created using Seurat’s *FindMarkers* function. Both functions apply the Wilcoxon rank sum test, a non-parametric statistical method. Differentially expressed genes (DEGs) between GG and non-GG^ctrl^ samples were identified using the *FindMarkers* function with a log fold-change threshold of 0.5, a minimum percent difference of 0.2, and an adjusted *P*-value cut-off of 0.05. Gene Ontology (GO) and the SynGO enrichment analysis were performed fisher’s exact test for statistical significance and correcting for multiple testing using the False Discovery Rate (FDR) method.

## Supplementary Material

fcaf372_Supplementary_Data

## Figures and Tables

**Figure 1:**
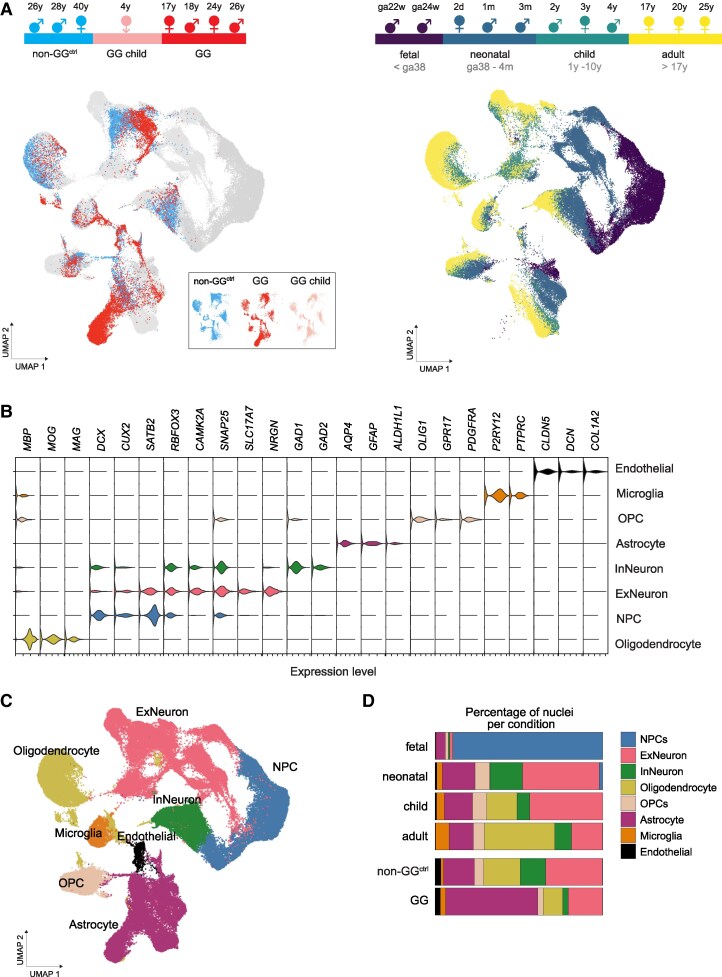
**Single-nucleus RNA sequencing analysis of GG reveals differences in transcriptomic landscape compared to various cortex controls.** (**A**) Uniform manifold approximation and projection (UMAP) distribution of single nuclei dissociated from 3 non-GG^ctrl^ (*n* = 9592), 4 GG adult (*n* = 11 377) and 1 GG child (*n* = 6445) tissues (left plot) superposed to the right UMAP plot. UMAP embedding of single nuclei from human cerebral cortex tissue coloured according to life stages: fetal (*n* = 22 457), neonatal (*n* = 28 679), child (*n* = 14 435) and adult (*n* = 22 453). Single-nucleus RNA-seq data of dataset 1 was downloaded from the GSE168408 provided by Herring *et al*., 2022.^20^ (right plot). Legends show the samples included in the study according to age and gender (left plot). ga, gastrulation age; GG, ganglioglioma. (**B**) Violin plots display the expression levels of canonical markers across the annotated cell types. The expression level was normalized by the ‘LogNormalize’ method in Seurat. Oligodendrocyte: *MBP* (Myelin Basic Protein), *MOG* (Myelin Oligodendrocyte Glycoprotein), *MAG* (Myelin-Associated Glycoprotein). Neuronal progenitor (NPC): *DCX* (Doublecortin), *CUX2* (Cut Like Homeobox 2), *SATB2* (SATB Homeobox 2). Neuron: *RBFOX3* (RNA Binding Fox-1 Homolog 3, also known as NeuN), *CAMK2A* (Calcium/Calmodulin Dependent Protein Kinase II Alpha), *SNAP25* (Synaptosome Associated Protein 25). Excitatory neuron (ExNeuron): *SLC17A7* (Solute Carrier Family 17 Member 7, also known as VGLUT1), *NRGN* (Neurogranin). Inhibitory neuron (InNeuron): *GAD1* (Glutamate Decarboxylase 1), *GAD2* (Glutamate Decarboxylase 2). Astrocyte: *AQP4* (Aquaporin 4), *GFAP* (Glial Fibrillary Acidic Protein), *ALDH1L1* (Aldehyde Dehydrogenase 1 Family Member L1). Oligodendrocyte precursor cell (OPC): *OLIG1* (Oligodendrocyte Transcription Factor 1), *GPR17* (G Protein-Coupled Receptor 17), *PDGFRA* (Platelet Derived Growth Factor Receptor Alpha). Microglia: *P2RY12* (Purinergic Receptor P2Y12), *PTPRC* (Protein Tyrosine Phosphatase Receptor Type C, also known as CD45). Endothelial: *CLDN5* (Claudin 5), *DCN* (Decorin), *COL1A2* (Collagen Type I Alpha 2 Chain). Statistical comparisons underlying the violin plots were performed using the non-parametric Wilcoxon rank sum test. (**C**) UMAP plot of all the nuclei under study clustered according to cell type. (**D**) Stacked bar graph of the proportion of nuclei assigned to each cell type across sample groups.

**Figure 2:**
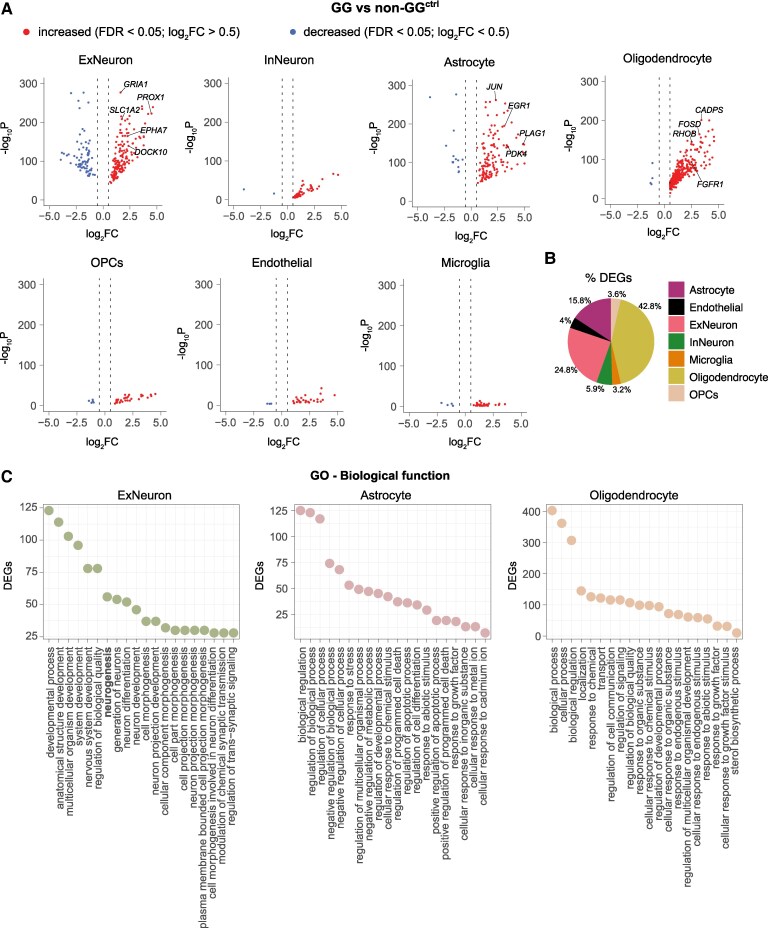
**Differential expression analysis of GGs reveals prominent transcriptomic alterations for excitatory neurons, oligodendroglia and astroglia.** (**A**) Volcano plot of the differentially expressed genes (DEGs) for each annotated cell type between GG and non-GG^ctrl^ groups. Note that the highest number of genes with differential expression is found in the excitatory neurons, oligodendrocytes and astrocytes cell types, for which key DEGs are labelled in the corresponding plots. Upregulated genes (increased, in *red*), *FDR* < 0.05 and *log_2_FC* > 0.5; downregulated genes (decreased, in *blue*), *FDR* < 0.05 and *log_2_FC* < 0.5. Min.diff.pct > 20%. (**B**) Pie chart of the fraction of DEGs for each cell type. (**C**) A dot plot displaying the 20 most significantly enriched terms from the Gene Ontology (GO) analysis, categorized by biological process, for excitatory neurons, astrocytes, and oligodendrocytes. GO analysis includes up- and down-regulated genes. Fisher’s exact test, with FDR correction. GG, ganglioglioma; FDR, False Discovery Rate; FC, fold-change; ExNeuron, Excitatory neuron; InNeuron, Inhibitory neuron; OPCs, Oligodendrocyte precursor cells.

**Figure 3:**
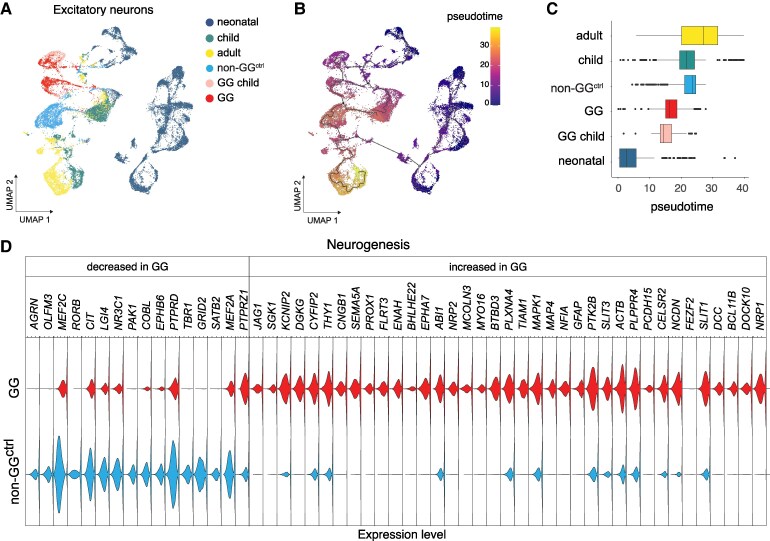
**Differential gene expression of excitatory neurons reveals a high overrepresentation of genes related to neuronal development.** (**A**) Uniform manifold approximation and projection (UMAP) distribution of the subset of nuclei assigned as excitatory neurons clustered according to the sample of origin. (**B**) Monocle pseudotime trajectory of excitatory neurons. UMAP distribution of excitatory neurons labelled by pseudotime values and representing their differentiation progression from dark blue (lowest) to yellow (highest). (**C**) Boxplot showing the distribution of pseudotime for each sample. Note the lower pseudotime level for GG compared to non-GG^ctrl^ samples. The pseudotime value of 0 represents the starting point of the trajectory (undifferentiated state): the pseudotime value of 40 represents the end of the trajectory (differentiated state). (**D**) Violin plot showing the expression level of the differentially expressed genes (DEGs) corresponding to the enriched Gene Ontology (GO) term ‘neurogenesis’. Statistical comparisons underlying the violin plots were performed using the non-parametric Wilcoxon rank sum test. GG, ganglioglioma.

**Figure 4:**
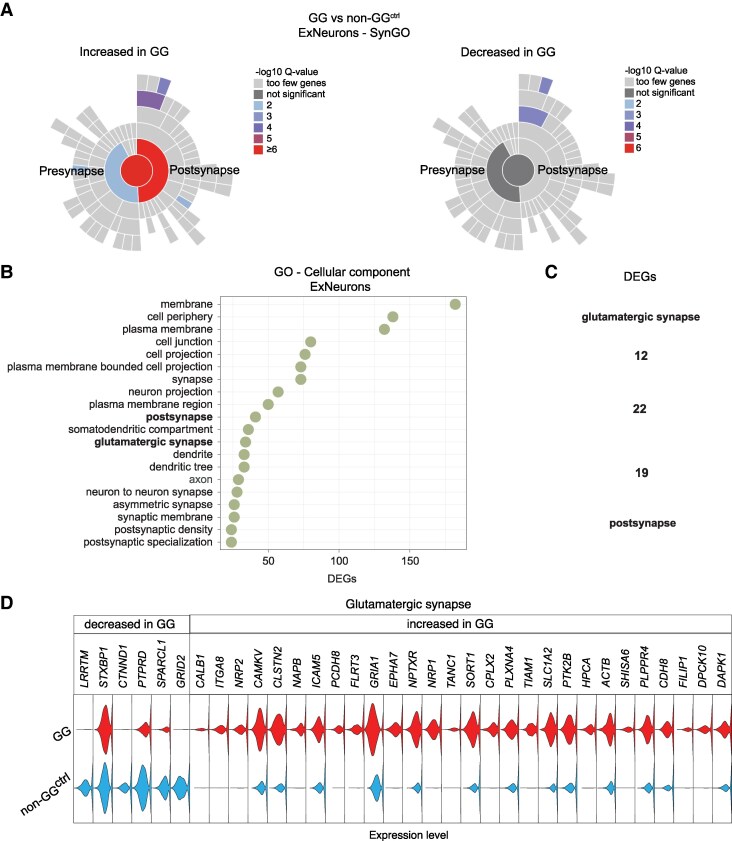
**Excitatory neurons are characterized by a fundamental augmentation of transcripts encoding postsynaptic proteins.** (**A**) Sunburst plots showing the SynGO annotation analysis of the top 60 most up- (left panel) and down-regulated (right panel) genes. Note the higher number of upregulated genes associated with the postsynaptic compartment. Legend colour shows the number of genes involved in each synaptic compartment. Fisher’s exact test, with FDR correction. (**B**) Dot plot showing the most enriched terms from the Gene Ontology (GO) analysis based on cellular components for excitatory neurons. Fisher’s exact test, with FDR correction (**C**) Ven diagram showing the level of overlap between the genes corresponding to ‘glutamatergic synapse’ and ‘postsynapse’ GO terms. (**D**) Violin plot showing the expression level of the differentially expressed genes (DEGs) corresponding to the ‘glutamatergic synapse’ GO term. Statistical comparisons underlying the violin plots were performed using the non-parametric Wilcoxon rank sum test. ExNeuron, Excitatory neuron, GG, ganglioglioma.

**Figure 5:**
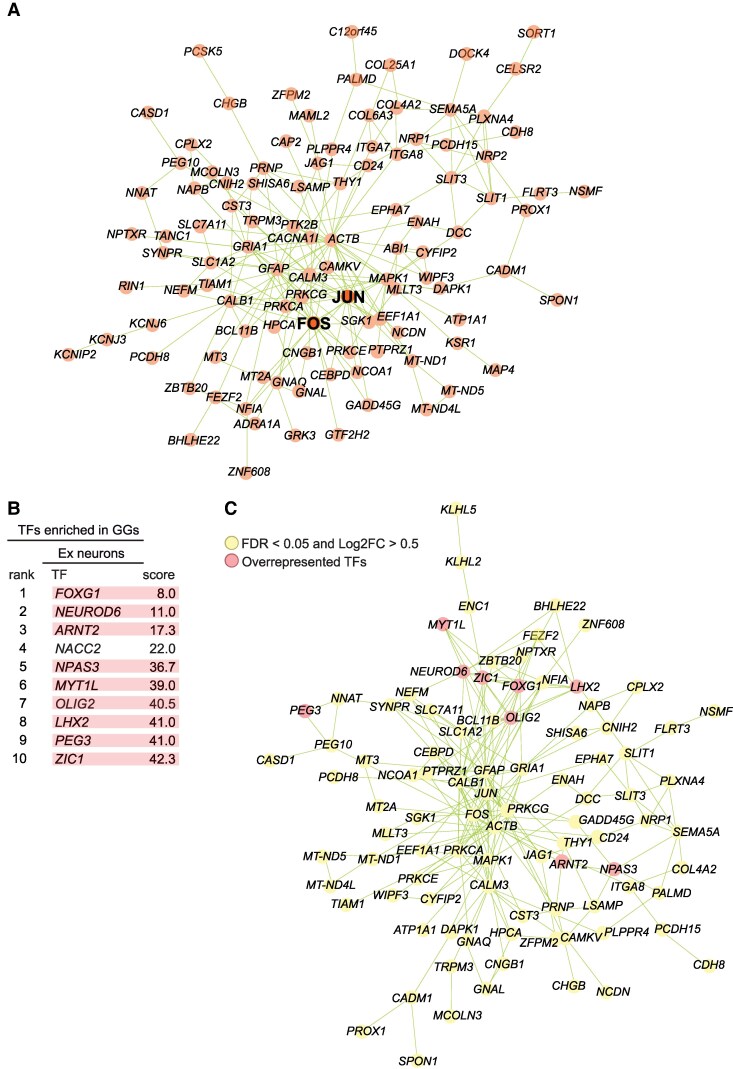
**Transcriptional hubs in GGs.** (**A**) STRING functional association network of differentially expressed genes of the excitatory neuron cluster (*FDR* < 0.05 and *Log_2_FC* > 0.5) with transcription factors (TFs) JUN and FOS centrally embedded. (**B**) TF enrichment of the differentially expressed genes of the excitatory neuron cluster. The 10 TFs with the highest score (based on ChEA 2022, ChEA Encode consensus, TTRUST 2019, JASPAR and TRANSFAC and Protein–Protein interaction) are given, with TF integrated into the STRING network (**A**). (**C**) Integration of the STRING network and ChEA database. FDR, False Discovery Rate; FC, fold-change; ExNeuron, Excitatory neuron. Fisher’s exact test, and TFs ranking based on the mean rank across all datasets.

## Data Availability

The data that support the findings of this study are available from the corresponding author upon request.

## Results

### Single-nucleus RNA sequencing analysis shows transcriptomic differences between GG and non-GG brain tissue

To study the transcriptomic and molecular architecture of human gangliogliomas (GGs), WHO grade 1, we followed the work-flow depicted in Supplementary Fig. 1. Five fresh frozen samples from different patients’ human GG tissue were obtained, including four samples from adults (from 17 to 26 years old) and one sample from a child (4 years old). All the brain specimens were from patients with a clinical history of epilepsy (Supplementary Tables 1 and 2). As controls, we used cryopreserved non-cancerous brain tissue from the temporal lobe (non-epileptic brain regions from patients with temporal lobe epilepsy; hereafter called non-GG^ctrl^; Fig. 1A, left panel, Supplementary Tables 1 and 2). After histological assessment confirming the diagnosis of GGs negative for BRAF^V600E^ (Supplementary Fig. 2), all samples were subjected to single-nucleus RNA sequencing (snRNA-seq), yielding 27 414 high-quality single nuclei transcriptome profiles after quality control and filtering (Supplementary Table 3). The resulting nuclei were bioinformatically merged with snRNAseq data from prefrontal cortex (PFC) nuclei of neurotypical individuals (GSE168408),^20^ including samples spanning different stages of development (from gastrulation to adult, Fig. 1A, right panel, Supplementary Table 3). Clustering analysis using Uniform Manifold Approximation and Projection (UMAP) revealed that certain nuclei clusters from GG adult samples exhibited a distinct transcriptomic profile compared to the non-GG^ctrl^ and PFC groups (Fig 1A). The clustering showed no striking differences between the GG adult and GG child samples (Fig. 1A, left panel, inset). However, as an attempt to compare the data to aged-grouped PFC cells, GG child sample was not considered for further downstream statistics-based analysis. Henceforth, we will refer to GG adult as GG throughout the manuscript.

Taking into account the levels of expression of canonical markers, we annotated each nucleus with a specific cell type; oligodendrocyte (*MBP*, *MOG*, *MAG*), neural precursor cell (NPC; *DCX*, *CUX2*, *SATB2*), excitatory neuron (ExNeuron; *RBFOX3*, *CAMK2A*, *SNAP25*, *SLC17A7*, *NRGN*), inhibitory neuron (InNeuron, *RBFOX3*, *CAMK2A*, *SNAP25*, *GAD1*, *GAD2*), astrocyte (*AQP4*, *GFAP*, *ALDH1L1*), oligodendrocyte precursor cell (OPC; *OLIG1*, *GPR17*, *PDGFRA*), microglia (*P2RY12*, *PTPRC*) and endothelial (*CLDN5*, *DCN*, *COL1A2*) (Fig. 1B and C). The quantitative analysis revealed a substantial rise in the proportion of astrocytes in the GG compared to the adult PFC and non-GG^ctrl^ samples (Fig. 1D). On the other hand, oligodendroglial cells and inhibitory neurons were slightly reduced in the GG compared to both adult PFC and non-GG^ctrl^ samples. Strikingly, the number of excitatory neurons in GG remained largely unchanged compared to both the adult PFC and non-GG^ctrl^ groups (Fig. 1D).

### Excitatory neurons, astrocytes and oligodendroglia show the highest degree of transcriptomic alterations in GGs

To follow up on the apparent molecular differences across cell types, we next aimed to determine the transcriptional changes in the individual cell types. For this purpose, we performed differential expression analysis for each cell type between human adult GG and non-GG^ctrl^ groups. Differentially expressed genes (DEGs) were filtered according to significance (*FDR* < 0.05) and change of expression (increase, *log_2_FC* > 0.5; decrease, *log_2_FC* < 0.5). Most DEGs were found for oligodendroglia (430 DEGs), excitatory neurons (250 DEGs) and astrocytes (159 DEGs). Fewer DEGs were observed within inhibitory neurons (57 DEGs), microglia (40 DEGs), endothelial (32 DEGs), and OPC (35 DEGs) nuclei populations (Fig. 2A). Accordingly, the excitatory neurons, oligodendroglia and astroglia populations are the cell types that mostly influence the overall transcriptomic changes in the GG group (Fig. 2B). Since the total number of nuclei was not the same among the individual cell types, we also tested for differential expression after normalization of the cells across cell types and found similar results (data not shown) demonstrating that the results are not biased by the different amounts of nuclei per cell type. We also performed an additional analysis using inferCNV to assess large-scale chromosomal alterations in excitatory neurons, astroglia and oligodendroglia in our single-nucleus RNA-seq dataset. Cells from the GG samples annotated as excitatory neurons, astroglia, and oligodendroglia were defined as the test (observation) groups. Endothelial cells from the same samples were used as the reference population, assuming a diploid genome. Our results showed that both neuronal as well as glial populations harbour genomic alterations across distinct chromosomes (Supplementary Fig. 5). This analysis demonstrates that both neuronal and glial populations within GGs harbour copy number variations consistent with neoplastic transformation, supporting the interpretation that these cells are part of the tumour clone.

We next sought to identify the functional profile of the cell type-specific DEGs of the GG and non-GG^ctrl^ samples. Given the large number of DEGs, we focused our functional analysis exclusively on excitatory neurons, astrocytes and oligodendrocytes. The DEGs identified across the three cell types were initially analysed for gene ontology (GO) enrichment to determine their associated biological functions. The genes differentially expressed in excitatory neurons showed a strong enrichment for GO-terms related to neuronal development and differentiation (e.g. ‘developmental process’, ‘neurogenesis’, ‘cell morphogenesis’ and ‘neuron differentiation’, Fig. 2C, left panel). In contrast, GO terms for pathways related to response to stimulus (e.g. ‘response to stress’, ‘response to growth factor’, ‘cellular response to metal ion’, ‘response to organic substance’) were found for the DEGs in both astrocytes and oligodendrocytes (Fig. 2C, middle and right panel). Strikingly, we also detected many GO pathways related to cell death for the astrocytes (Fig. 2C, middle panel). GO analysis for the molecular function revealed that astrocytes and oligodendrocytes have a strong enrichment for genes related to transcription factor activity, purine nucleotide and ribonucleotide binding (Supplementary Fig. 3). The GO analysis results point to a combination of developmentally abnormal excitatory neurons together with metabolically active/responsive astrocyte and oligodendrocyte cell populations.

### Excitatory neurons from GG tissue show a more immature phenotype compared to non-GG controls

The enrichment of developmental-related pathways observed in excitatory neurons prompted us to further investigate their differentiation status through trajectory analysis. First, the excitatory neurons from all samples, including the nuclei from the PFC dataset (GSE168408,^20^ Fig. 1A), were isolated and re-clustered. Secondly, we conducted a trajectory analysis to monitor the developmental progression, using the NPCs from the PFC dataset as the most undifferentiated cells (Fig. 3A). The excitatory neurons from the non-GG^ctrl^ group had a similar level of maturation compared to the excitatory neurons from the PFC dataset. Strikingly, excitatory neurons of the GG group showed lower levels in pseudotime than the non-GG^ctrl^ group and PFC datasets from adult and child groups, but higher levels compared to the neonatal PFC dataset (Fig. 3B and C). This highlights a unique and more immature stage of excitatory neurons in the GG samples. Finally, to get information on the individual genes potentially influencing this state of activation, we assessed the expression level of the GO pathway ‘neurogenesis’. We observed that the expression of 69.6% of the genes was increased in the GG group compared to the non-GG^ctrl^ group (Fig. 3D). Among the set of genes associated with brain development, *SLIT1/3*, *SEMA5A*, and *EPHA7* were found to have elevated expression levels in GG, while genes such as *MEF2A/C* and *MEF2A* showed reduced expression in GG compared to non-GG^ctrl^. Since immature features have been already described in the context of BRAF^V600E^-positive GGs, we assessed transcriptomic differences in excitatory neurons between BRAF^V600E^-positive and -negative gangliogliomas (GGs), we analysed a public snRNA-seq dataset including four BRAF^V600E^-positive GGs. Neuronal cells were annotated using canonical markers and classified as excitatory or inhibitory. We combined excitatory neurons from both groups for joint analysis and performed differential gene expression analysis. The results revealed partially overlapping but distinct transcriptional profiles, reflecting the different molecular contexts associated with BRAF^V600E^ mutation (Supplementary Fig. 6A–D). GO enrichment analysis of the top upregulated genes in each group showed functional convergence, with both neuron types enriched in pathways related to nervous system development and synaptic signalling (Supplementary Fig. 6E). This suggests that while the specific molecular drivers may differ, excitatory neurons in both tumour subtypes engage similar developmental and functional programmes.

### Excitatory neurons in BRAF^V600E^-negative GG show increased expression levels of transcripts related to synaptic function

Because of the potential contribution of excitatory neurons in neuronal excitability, we next investigated the extent of differential expression of genes related to the synapse. We submitted DEGs to the neuronal enrichment analysis SynGO.^23^ To compare the contribution of increased and decreased transcripts, we submitted the same number of genes for both groups (top 60 DEGs per group). SynGO analysis revealed that more synaptic genes were found in the DEGs that were increased in the GG group (30%) compared to the DEGs that were decreased in GGs (15%) (Fig. 4A). Interestingly, among the increased DEGs in the GG group, the SynGO annotation ‘postsynapse’ appeared as the GO term with the highest enrichment score.

We further analysed the DEGs with GO enrichment analysis for the cellular component of excitatory neurons. The corresponding results revealed a high enrichment for the GO annotation ‘cell membrane’, ‘cell periphery’ or ‘cell junction’ (Fig. 4B) suggesting the involvement of the neurons in processes such as signalling, synaptic communication and structural integrity. Strikingly, our data depicted a high enrichment for glutamatergic synapse transmission, which included 34 DEGs (Fig. 4B). From these genes classified as glutamatergic, 22 (64.7%) were assigned as postsynaptic (Fig. 4C), which aligns with the findings from the SynGO analysis. Notably, a significant proportion of glutamatergic genes (82.3%) showed increased expression in GG compared to non-GG^ctrl^ samples: among others, *GRIA1*, *SLC1A2, PTK2B* or *SHISHA6* (Fig. 4D).

Collectively, these findings demonstrate that excitatory neurons in GG tissue exhibit elevated expression of genes associated with postsynaptic glutamatergic transmission.

### Transcriptional hubs as potential therapeutic targets for GGs

We next sought to identify key transcriptional networks that could serve as potential hubs for therapeutic intervention. To investigate whether the transcriptional profiles of GGs are regulated by specific transcription factors (TFs), we first assembled all DEGs of the excitatory neuron cluster (*FDR* < 0.05 and *Log_2_FC* > 0.5) into a STRING functional association network.^22^ Interestingly, at the centre of the network, two transcription factors—namely the immediate early genes (IEGs) *JUN* and *FOS*—are embedded (Fig. 5A, Supplementary Fig. 4), This finding potentially indicates that these TFs act as transcriptional hubs in GG regulation.

As a complementary approach, we performed a TF enrichment analysis (TFEA) to test whether the DEGs in the excitatory neurons have overrepresented TF binding sites (TFBS) for specific TFs. This analysis revealed strong enrichment for several TFs, including the TFs Forkhead Box G1 (*FOXG1*), Neuronal Differentiation 6 (*NEUROD6*), Aryl Hydrocarbon Receptor Nuclear Translocator 2 (*ARNT2*), oligodendrocyte transcription factor 2 (*OLIG2*), and Zinc finger of the cerebellum 1 (*ZIC1*) (Fig. 5B). When integrating the top 10 overrepresented TFs into the excitatory network, we observed that all TFs, except for Nucleus Accumbens Associated 2 (*NACC2*), were connected within the web. Interestingly, *OLIG2*, *FOXG1* and *ZIC1* appeared to integrate most strongly within the network (Fig. 5C), suggesting that these TFs could serve as potential therapeutic targets for GGs.

## Discussion

The high percentage of GG WHO grade 1 positive for BRAF^V600E^ warrants further research as patients may benefit from clinically available BRAF and MEK inhibitors (e.g. vemurafenib).^24,25^ However, several reports demonstrated no significant difference in seizure outcomes between GGs with or without the mutation.^6,17,18^ In our transcriptomic study, we included patients with BRAF^V600E^-negative tumours fulfilling histological and immunohistochemical criteria of GGs and a clinical history of epilepsy to identify novel molecules underlying neuronal excitability independently of the mutation. While the inclusion of pharmacologically treated patients introduces some variability in the molecular signatures observed, this reflects the clinical heterogeneity of real-world epilepsy care. As such, our findings may be particularly relevant for understanding disease mechanisms in typical treatment contexts.

Consistent with previous studies suggesting developmental impairments in neurons within GGs,^26^ we identified excitatory neurons with a less mature transcriptomic profile compared to those in control tissue in BRAF^V600E^-negative GGs. This finding reinforces the concept recently highlighted for BRAF^V600E^-positive GGs using single-cell transcriptomics.^12^ Our results reveal transcriptomic alterations in several genes central to brain developmental programmes. Among these, we found transcriptomic alterations in *SEMA5A*, for which mutations in early-onset epileptic encephalopathy have been reported,^27^  *TIAM1*^28^ and genes of the MEF2 family.^29^ Moreover, we also examine a publicly available dataset on BRAF^V600E^-positive GGs and show transcriptomic alterations related to developmental pathways in excitatory neurons. These findings support the notion that BRAF^V600E^-associated transcriptional divergence may converge on shared neurodevelopmental outcomes, potentially through distinct regulatory mechanisms.

The transcriptomic signatures of neuronal immaturity contrast with previous data demonstrating a premature neuronal maturation triggered by BRAF^V600E^-expressing murine GG cells.^30^ Although these findings arise from different methodologies—functional analysis versus transcriptomic profiling—they suggest that distinct mechanisms independent of MAPK pathway activation underlie the neuronal immaturity observed in BRAF^V600E^-negative GG.

Surprisingly, the expression of the oncofoetal gene CD34, which is often highly expressed in GGs,^31,32^ was primarily restricted to endothelial cells in all samples, even though the immunochemical assessment showed strong expression in 75% of BRAF^V600E^-negative GGs (Supplementary Table 1). A recent study has demonstrated increased CD34 expression in astrocyte/like clusters in BRAF^V600E^-positive GGs.^12^ This suggests that elevated CD34 levels in BRAF^V600E^-negative GGs may arise through distinct mechanisms, likely independent of transcriptomic regulation.

Along with an immature cellular profile, the excitatory neuronal compartment in GGs exhibited stronger expression of synaptic genes compared to non-GG control tissue, most of which were classified as postsynaptic. Genes associated with glutamatergic transmission, such as *GRIA1*, *CAMKV*, *NPTXR* or *SHISA6*, were among the most enriched. This phenomenon together with previous studies showing increased extracellular glutamate in the media of cultures of mouse GG cells^30^ and alterations in glutamate receptors within GG parenchyma^33^ suggests that glutamatergic transmission may be a central mechanism driving neuronal excitability in this tumour entity. Notably, the increased expression of postsynaptic genes was exclusive for excitatory neurons, potentially contributing to changes in the overall neuronal network. Hence, this study provides clear evidence that genetic alterations, independent of the BRAF^V600E^ variant, cause a more immature phenotype of excitatory neurons and exhibit a greater number of transcriptomic alterations in the synaptic compartment compared to neurons in the non-GG control tissue.

In contrast, no significant changes in gene expression were observed in inhibitory neurons, suggesting that this cell type may not directly contribute to neuronal excitability through transcriptomic alterations. Interestingly, we observed a reduced number of inhibitory neurons in human GGs, consistent with previous findings showing a reduction in the adjacent cortical areas of human GGs^34^ and in mouse models.^35^ This suggests that increased excitation may be further supported by a reduced inhibitory neuronal population. Our findings overall support the hypothesis that GGs likely originate from a pluripotent precursor in the paraventricular zone, capable of differentiating into multiple cellular lineages, including glial and excitatory neurons. The relative paucity of inhibitory interneurons may indicate an unfavourable microenvironment in the GGs as destination for tangentially migrating interneuronal precursors, which may be at least in part due to an immature excitatory neuronal network or non-physiological non-neuronal cells such as aberrantly differentiated astroglia.

Assessment of cell type architecture revealed an increased number of astrocytes in GGs compared to non-GG controls. Moreover, astrocytes (and oligodendrocyte-like cells) in GGs exhibit significant transcriptomic changes. However, their associated functions are more generalist and broad; pathways related to regulation, maintenance, and stress response suggest a homeostatic role, possibly reflecting their neoplastic nature. In BRAF^V600E^-mutant GGs, glial cells have been linked to tumour recurrence and aggressive outcomes.^36^ Moreover, astrocytic and oligodendroglial differentiation has been reported to be driven by BRAF and FGFR1 mutations in GG and dysembryoplastic neuroepithelial tumours, respectively.^37^ Thus, the lack of BRAF^V600E^ in GG could also contribute to a unique neoplastic profile.

Interestingly, microglial cell numbers remained unchanged. This might be explained by the absence of the BRAF^V600E^ mutation, as this mutation has been implicated in immune regulation/activation in GGs^7,38^ and other tumours such as pleomorphic xanthoastrocytoma^39^ and melanomas.^40,41^ Moreover, recent findings have also associated KIAA1549-BRAF mutation with high expression of immune-related chemokines in pilocytic astrocytomas,^42^ suggesting immune-related dependence on MAPK overactivation cascade. As all patients included in this study had epilepsy, our data demonstrates that the epilepsy phenotype is independent of microglial reactivity in BRAF^V600E^-negative tumours.

Our study identified transcriptional factors within the BRAF^V600E^-negative GGs that could serve as targets for therapeutic intervention. *JUN* and *FOS* emerged as central TFs, highlighting their potential role as transcriptional hubs in the regulatory network. *JUN* and *FOS* are well-documented as IEGs involved in cellular responses to various stimuli, including stress, inflammation, and neuronal activity (reviewed by^43,44,45^). Their roles as TFs are particularly significant in the context of neural development, plasticity, and pathological conditions like cancer,^42,46-49^ making them perfect candidates for further exploration in GG targeted therapies.

Besides these two IEGs, TFs such as *OLIG2*, *FOXG1* and *ZIC1* appeared to integrate most strongly within the network. *OLIG2*, a TF primarily associated with neural progenitor cells and the development of oligodendrocytes in the central nervous system, has been shown to play a role in several types of brain tumours, including gliomas. In these tumors, OLIG2-positive cells show heterogeneous characteristics, sometimes exhibiting both glial and neuronal features.^50-53^ However, its exact role in GG pathogenesis and progression remains an area for further research. Although no direct evidence exists linking *FOXG1* expression to GGs specifically, *FOXG1* has been shown to play a crucial role in brain tumour development and progression and plays a significant role in the more aggressive type of brain tumor glioblastoma multiforme (GBM).^54^ ZIC1 has been implicated in various brain tumors and cancers, including low-grade gliomas,^55,56^ suggesting a role in tumorigenesis. Further research would be necessary to confirm and expand their relevance for therapeutic targeting of GGs.

Overall, our study suggests that epileptogenicity in BRAF^V600E^-negative GGs may result from the presence of immature, aberrantly connected excitatory neurons, a relative deficiency of inhibitory neurons, and dysfunctional astro-/oligodendroglial cells with altered metabolic activity. Addressing refractory epilepsy is one of the major challenges in managing GGs. Therapeutic stratification based on BRAF^V600E^ mutation status may guide treatment approaches. Our findings open avenues for new therapies, suggesting that targeting genes to both development and synaptic processes in excitatory neurons within the tumour may provide a basis for effective combinatorial treatments.

## Data availability

The sequencing data generated for this study have been deposited in the NCBI Sequence Read Archive (SRA) under accession number SUB15314645. The code generated for this work has been submitted as Supplementary Table 5.
