# Supplementary material for: Single-nucleus RNA sequencing: immature excitatory neurons and transformed glia build human BRAFV600E-negative gangliogliomas
Source: Brain Commun. 2025 Oct 1;7(5):fcaf372. doi: 10.1093/braincomms/fcaf372 (PMC12516308; doi:10.1093/braincomms/fcaf372)
Supplement: fcaf372_Supplementary_Data [file fcaf372_supplementary_data.docx]

Supplementary Data

# Content

[Content 1](#_Toc209350441)

[Supplementary Figures 2](#_Toc209350442)

[Supplementary Figure 1 2](#_Toc209350443)

[Supplementary Figure 2 3](#_Toc209350444)

[Supplementary Figure 3 5](#_Toc209350445)

[Supplementary Figure 4 6](#_Toc209350446)

[Supplementary Figure 5 7](#_Toc209350447)

[Supplementary Figure 6 8](#_Toc209350448)

[Supplementary Tables 10](#_Toc209350449)

[Supplementary Table 1 10](#_Toc209350450)

[Supplementary Table 2 11](#_Toc209350451)

[Supplementary Table 3 12](#_Toc209350452)

[Supplementary Table 4 13](#_Toc209350453)

[Supplementary Table 5 16](#_Toc209350454)

# Supplementary Figures

## Supplementary Figure 1


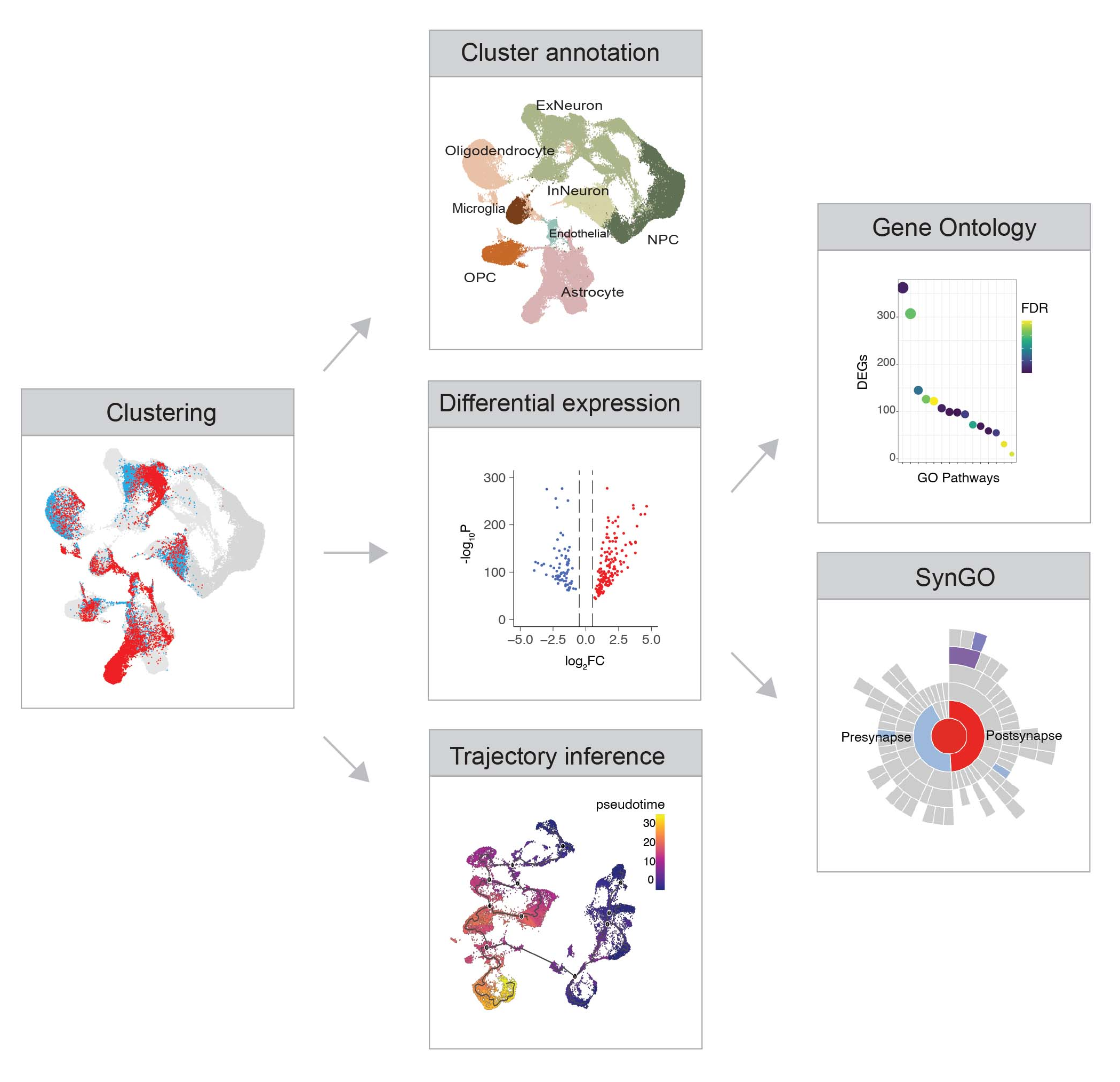


**Supplementary Figure 1 Scheme of the experimental design for the analysis of single nucleus RNA sequencing data.** The experimental workflow begins with the clustering of the sequenced nuclei, which enables cell-type annotation, differential gene expression analysis and cell-type-specific trajectory inference. Additionally, gene expression changes are subjected to gene ontology enrichment analysis to identify biological pathways associated with specific cell types and SynGO analysis to investigate the expression of synapse-related genes.

## Supplementary Figure 2


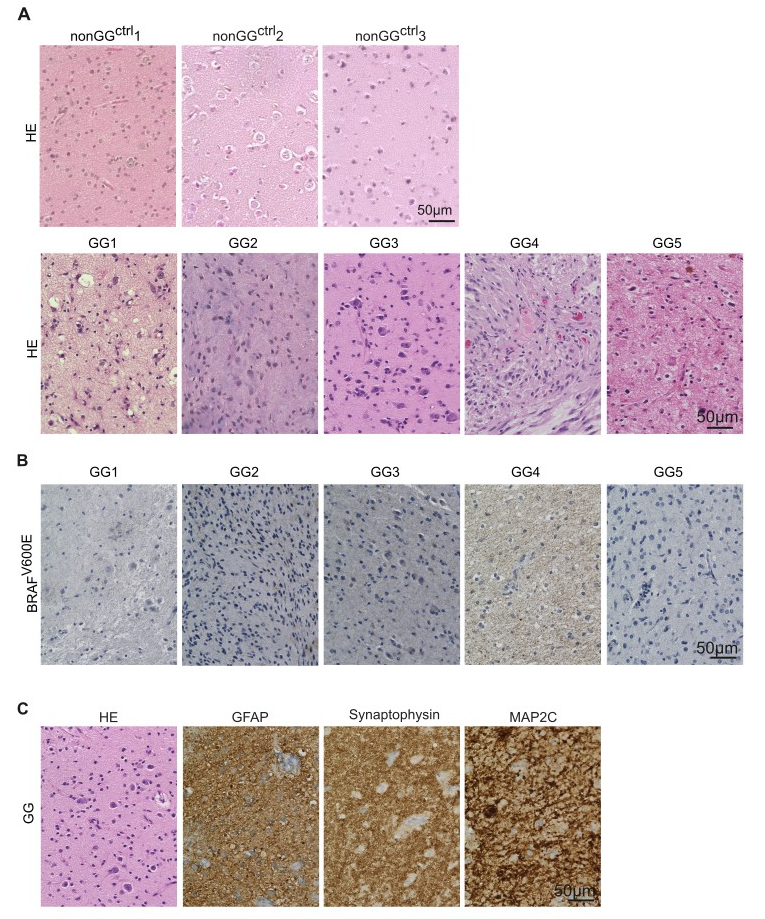


**Supplementary Figure 2 Histological assessment of the non-GG^ctr^ and GG samples and BRAF^V600E^ immunohistochemical analysis of GG samples.** (**A**) Representative images from hematoxylin and eosin-stained non-GG^ctr^ and GG brain slices. (**B**) Representative images from BRAF^V600E^-stained GG sections confirming the negative status for BRAF^V600E^ for all GG cases. Each image corresponds to a different biological replicate for both groups. (**C**) Representative images from GG tissue immunolabeled with antibodies against GFAP (astrocytic component), Synaptophysin and MAP2C (neuronal component). Images from immunolabeled tissue were taken from sample GG4. Scale bar, 50 μm.

## Supplementary Figure 3


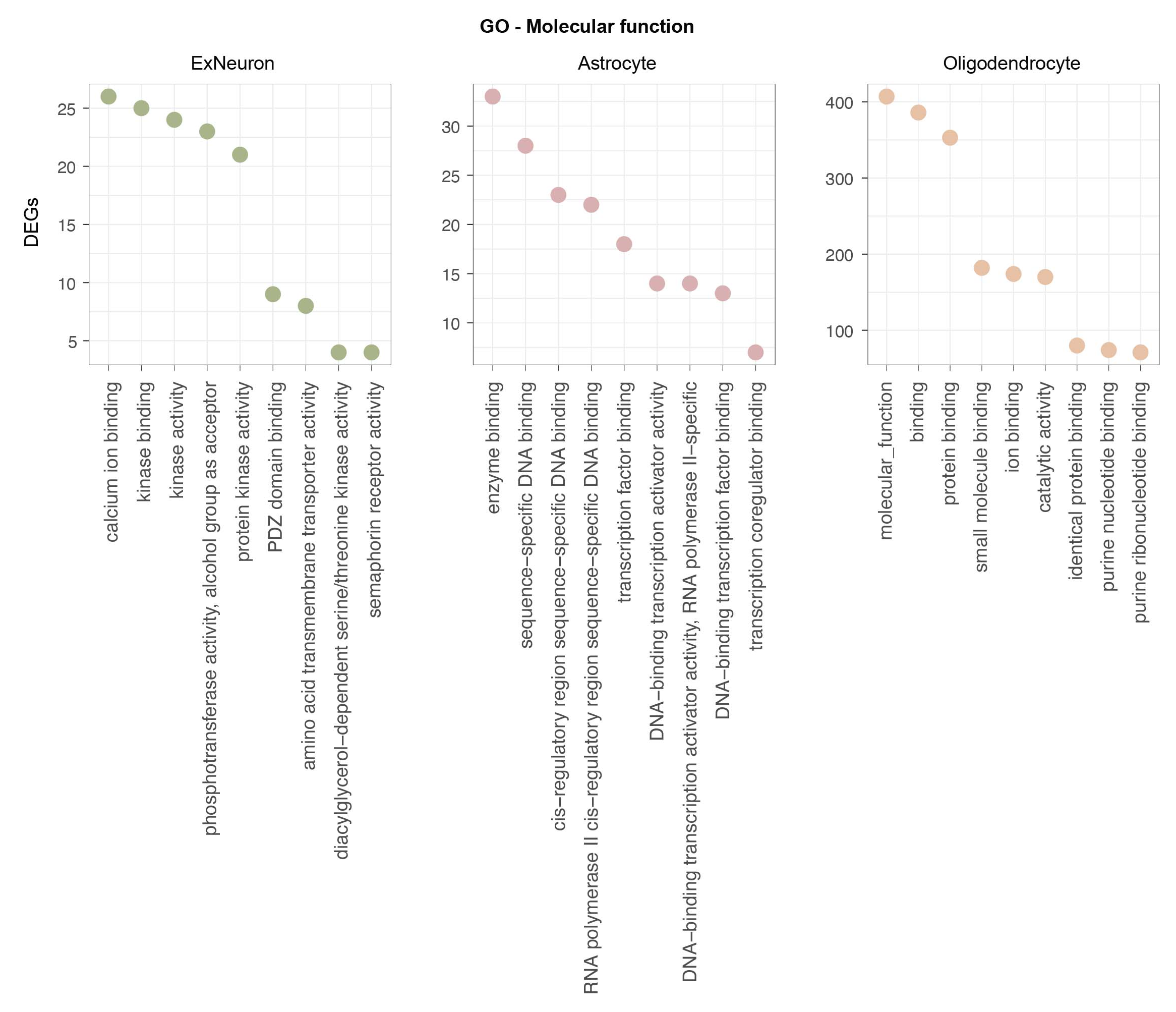


**Supplementary Figure 3 Gene Ontology (GO) enrichment analysis of the molecular function from differentially expressed genes in neurons, astrocytes and oligodendrocytes.** Dot plots showing the 15 most differentially expressed functional pathways, categorized by molecular function, for excitatory neurons (left graph), astrocytes (middle graph), and oligodendrocytes (right graph). GO analysis includes up- and down-regulated genes. Fisher’s exact test, with False Discovery Rate (FDR) correction. All functional pathways show an FDR < 0.05.

## Supplementary Figure 4


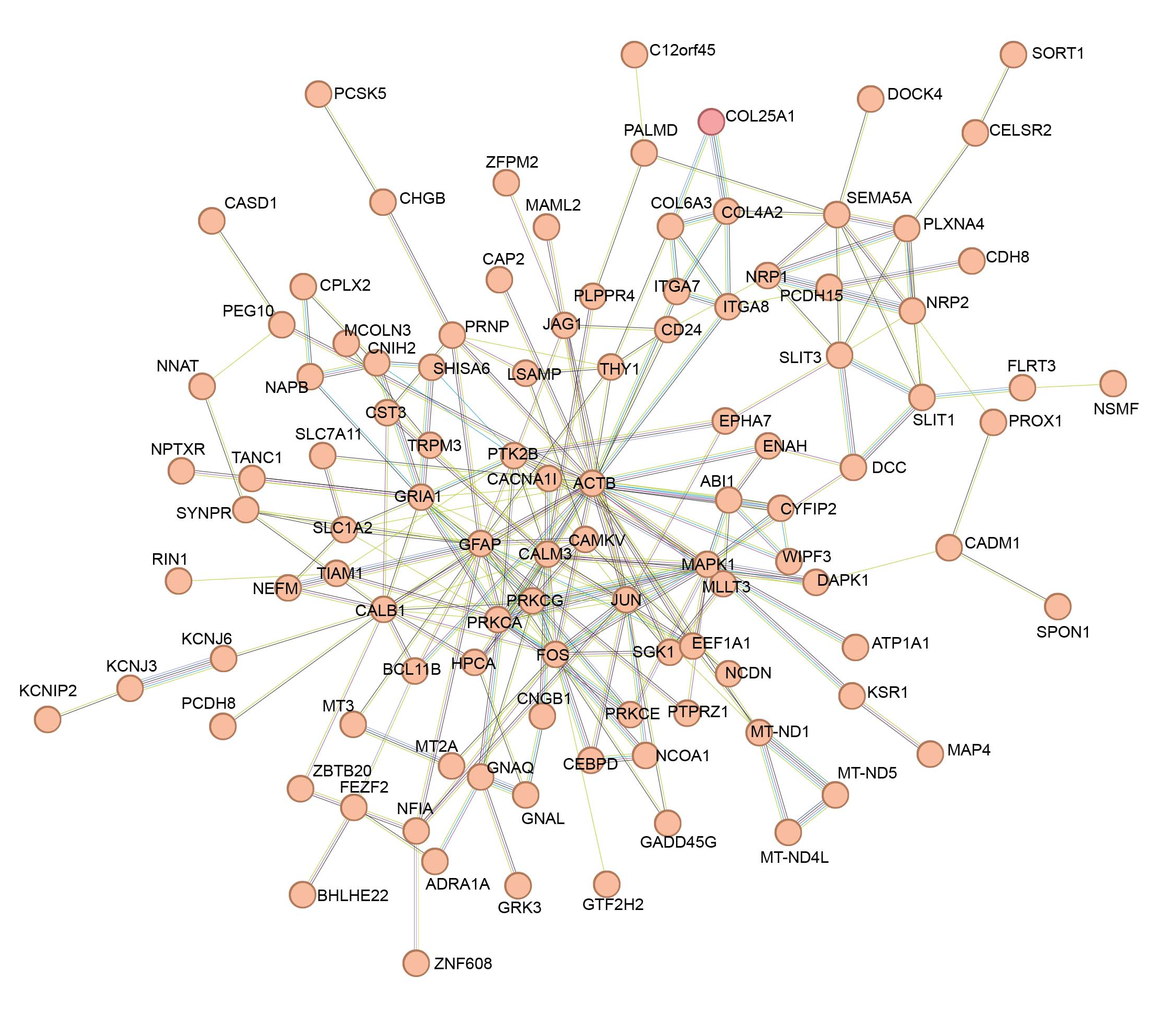


**Supplementary Figure 4 STRING Network of Differentially Expressed Genes in Excitatory Neurons.** Functional association network from STRING analysis, highlighting differentially expressed genes (FDR < 0.05, Log_2_FC > 0.5) within the excitatory neuron cluster. The transcription factors JUN and FOS are centrally positioned, suggesting a key regulatory role in the network.

## Supplementary Figure 5


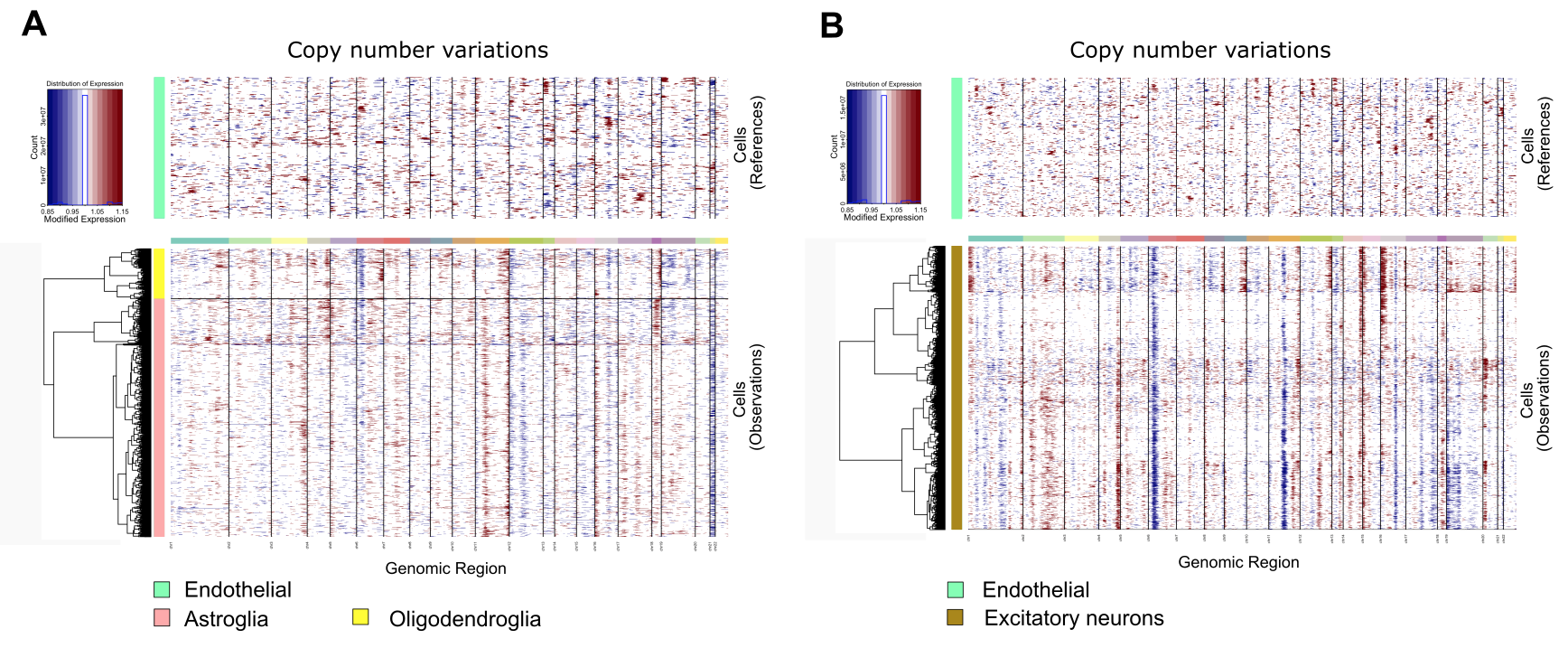


**Supplementary Figure 5 Copy number alteration analysis of excitatory neurons, astroglia and oligodendroglia in gangliogliomas.** Estimation of copy number variants by inferCNV in astroglia and oligodendroglia (**A**) and excitatory neurons (**B**). Large-scale CNV patterns are shown for glial and neuronal populations within the ganglioglioma sample. Upper heatmap corresponds to the reference cells (endothelial) and the lower heatmap corresponds to the observation groups: astroglia/oligodendroglia (**A**) and excitatory neurons (**B**). The top color bars indicate distinct chromosome regions. The colors represent relative expression levels of genes across the genome, used as a proxy to infer CNV in each cell. Red or blue indicates higher (possible amplification) or lower (possible deletion) values compared with the reference cells. The analysis was run with a gene expression cutoff of 0.1, denoising enabled, and HMM-based CNV prediction (HMM type = i6).

## Supplementary Figure 6


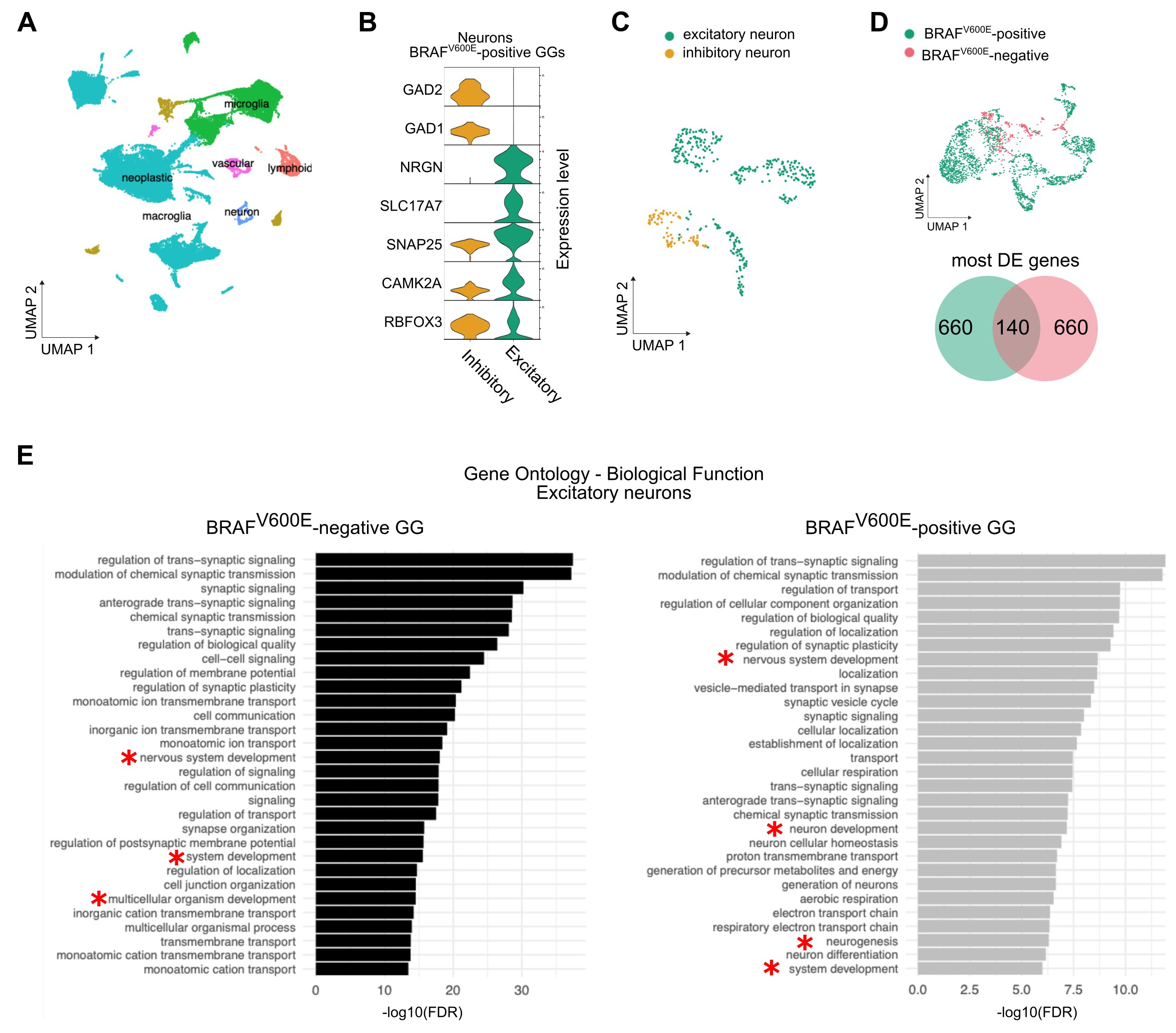


**Supplementary Figure 6 Transcriptional and functional analysis in excitatory neurons from GGs with and without BRAF^V600E^.** (**A**) UMAP visualization of single-nucleus RNA-sequencing data from BRAF^V600E^-positive gangliogliomas, with major cell types annotated. (**B**) Violin plots of selected marker genes distinguishing excitatory (e.g., NRGN, SLC17A7) and inhibitory (e.g., GAD1, GAD2) neurons, confirming neuronal subpopulation identity. (**C**) UMAP of re-clustered neurons from BRAF^V600E^-positive GGs colored by excitatory (green) and inhibitory (orange) identity. (**D**) UMAP of BRAF^V600E^-positive and BRAF^V600E^-negative excitatory neurons showing distinct transcriptomic profiles (upper panel). Venn diagram indicates the number of differentially expressed genes (DEGs) unique to each group and shared (lower panel). (**E**) Bar graphs of the most significantly enriched Gene Ontology biological processes from the most 800 differentially expressed genes for excitatory neurons from BRAF^V600E^-negative (left, black) and BRAF^V600E^-positive (right, grey) gangliogliomas. Bars represent –log10(FDR); red asterisks indicate processes related to neuronal development. GO analysis includes up- and down-regulated genes. Fisher’s exact test, with False Discovery Rate (FDR) correction. All functional pathways show an FDR < 0.05.

# Supplementary Tables

## Supplementary Table 1

**Supplementary Table 1 Histopathological information on the human GGs and non-GGctrl samples used in this study.**

| GG sample | Age (years) | Gender | Localization | CD34 | Ki67 | Clinical presentation |
| --- | --- | --- | --- | --- | --- | --- |
| 1 | 17 | Female | Right TL | Strong expression | 2 % | focal seizures (simple / complex) |
| 2 | 26 | Male | Left TL | Strong expression | Spare | focal seizures (complex), FBTCS |
| 3 | 4 | Female | Left TL | Negative expression | <2 % | focal seizures (simple / complex) |
| 4 | 24 | Female | Left TL | Spare expression | 5 % | seizures, therapy-refractory |
| 5 | 17 | Male | Right TL | Strong expression | 3 % | focal seizures (complex), FBTCS |

| **Non-GGctrl sample** | **Age (years)** | **Gender** | **Diagnosis** | **Localization** | **Clinical presentation** |
| --- | --- | --- | --- | --- | --- |
| 1 | 37 | Female | Hippocampal sclerosis | Left TL | focal seizures (complex) |
| 2 | 45 | Male | Hippocampal sclerosis | Left TL | focal seizures (complex), FBTCS |
| 3 | 24 | Female | Hippocampal sclerosis | Right TL | focal seizures (simple/complex), FBTCS |

TL: temporal lobe

FBTCS: focal to bilateral tonic clonic seizure

## Supplementary Table 2

Supplementary Table 2 Pharmacological treatments administered to human ganglioglioma (GG) and non-GG control patients included in this study. Note that none of the patients underwent any prior brain surgery before GG or hippocampal sclerosis resection.

| GG sample | Clinical Presentation | Pharmacotherapy  Pre-surgery | Pharmacotherapy  Post-surgery |
| --- | --- | --- | --- |
| 1 | focal seizures  (simple / complex) | Clonazepam, Lamotrigine | Clonazepam, Lamotrigine  (for 2 years post-surgery)  Brivaracetam  (from 15 years post-surgery on) |
| 2 | focal seizures (complex), FBTCS | Barbexaclone, Carbamazepine, Lamotrigine, Valproic Acid, Phenytoin, Topiramate, Oxcarbazepine | Oxcarbazepine  (for 3 years after surgery),  Lamotrigine  (from 2 years post-surgery on) |
| 3 | focal seizures  (simple / complex) | Sulthiame,  Lamotrigine | Lamotrigine |
| 4 | seizures,  therapy-refractory | Carbamazepine,  Levetiracetam | Carbamazepine  (for 2 years post-surgery),  Levetiracetam  (from 1 year post-surgery on) |
| 5 | focal seizures (complex), FBTCS | Levetiracetam,  Oxcarbazepine | Levetiracetam (for 5 years post-surgery),  Oxcarbazepine  (for 3 years post-surgery) |
| Non-GG control sample | Clinical Presentation | Pharmacotherapy  Pre-surgery | Pharmacotherapy  Post-surgery |
| 1 | focal (complex) seizures | Topiramate, Brivaracetam | Topiramate, Brivaracetam  (for 9 months post-surgery) |
| 2 | focal (complex) and bilateral tonic-clonic seizures | Valproic acid, Brivaracetam | Brivaracetam,  Valproic acid  (for 3 months post-surgery),  Lacosamide  (from 3 months post-surgery on) |
| 3 | focal (simple/complex) and bilateral tonic-clonic seizures | Lamotrigine, Topiramate | Lamotrigine  (for 30 months post-surgery),  Topiramate  (for 20 months post-surgery) |

FBTCS: focal to bilateral tonic clonic seizure

## Supplementary Table 3

**Supplementary Table 3 Information about the samples used for snRNAseq, including the experimental groups, the GEO number and the total number of single nuclei analyzed.**

| **Sample name** | **Group** | **Gender** | **GEO number** | **Number of cells after filtering** |
| --- | --- | --- | --- | --- |
| ga22 | Fetal | M | GSE168408 | 11660 |
| ga24 | Fetal | M | GSE168408 | 10797 |
| pn1m | Neonatal | M | GSE168408 | 11034 |
| pn2m | Neonatal | M | GSE168408 | 7968 |
| pn3m | Neonatal | M | GSE168408 | 9677 |
| pn2y_b | Child | M | GSE168408 | 2410 |
| pn3y | Child | F | GSE168408 | 3555 |
| pn4y | Child | M | GSE168408 | 8470 |
| pn17y_b | Adult | F | GSE168408 | 8014 |
| pn20y_a | Adult | F | GSE168408 | 7382 |
| pn25y_b | Adult | F | GSE168408 | 7057 |
| GG1 | GG_adult | F | Cases-Cunillera et al., | 6657 |
| GG2 | GG_adult | M | Cases-Cunillera et al., | 140 |
| GG3 | GG_child | F | Cases-Cunillera et al., | 6445 |
| GG4 | GG_adult | F | Cases-Cunillera et al., | 974 |
| GG5 | GG_adult | M | Cases-Cunillera et al., | 3606 |
| nonGGctrl1 | nonGGctrl_adult | F | Cases-Cunillera et al., | 3875 |
| nonGGctrl2 | nonGGctrl_adult | M | Cases-Cunillera et al., | 2850 |
| nonGGctrl3 | nonGGctrl_adult | F | Cases-Cunillera et al., | 2867 |

ga: gestational age

pn: postnatal

m: months

y: year

## Supplementary Table 4

**Supplementary Table 4 Full names of gene symbols displayed in Figures and Supplementary figures.**

| **Gene symbol** | **Gene full name** |
| --- | --- |
| ABI1 | Abl Interactor 1 |
| ACTB | Actin Beta |
| ADRA1A | Adrenoceptor Alpha 1A |
| AGRN | Agrin |
| ALDH1L1 | Aldehyde Dehydrogenase 1 Family Member L1 |
| AQP4 | Aquaporin 4 |
| ARNT2 | Aryl Hydrocarbon Receptor Nuclear Translocator 2 |
| ATP1A1 | ATPase Na+/K+ Transporting Subunit Alpha 1 |
| BCL11B | BAF Chromatin Remodeling Complex Subunit BCL11B |
| BHLHE22 | Basic Helix-Loop-Helix Family Member E22 |
| C1orf61 | Chromosome 1 Open Reading Frame 61 |
| CALB1 | Calbindin 1 |
| CALM3 | Calmodulin 3 |
| CAMK2A | Calcium/Calmodulin Dependent Protein Kinase II Alpha |
| CAMKV | Calmodulin Kinase Like Vesicle Associated |
| CAP2 | Cyclase Associated Actin Cytoskeleton Regulatory Protein 2 |
| CASD1 | CAS1 Domain Containing 1 |
| CD24 | CD24 Molecule |
| CEBPD | CCAAT Enhancer Binding Protein Delta |
| CELSR2 | Cadherin EGF LAG Seven-Pass G-Type Receptor 2 |
| CHGB | Chromogranin B |
| CLPB | Caseinolytic Mitochondrial Matrix Peptidase Chaperone Subunit B |
| CNGB1 | Cyclic Nucleotide Gated Channel Beta 1 |
| CNIH2 | Cornichon Family AMPA Receptor Auxiliary Protein 2 |
| COL1A2 | Collagen Type I Alpha 2 Chain |
| COL25A1 | Collagen Type XXV Alpha 1 Chain |
| COL4A2 | Collagen Type IV Alpha 2 Chain |
| COL6A3 | Collagen Type VI Alpha 3 Chain |
| CPLX2 | Complexin 2 |
| CST3 | Cystatin C |
| CTNND1 | Catenin Delta 1 |
| CUX2 | Cut Like Homeobox 2 |
| CYFIP2 | Cytoplasmic FMR1 Interacting Protein 2 |
| DAPK1 | Death Associated Protein Kinase 1 |
| DCC | Deleted in Colorectal Carcinoma |
| DCN | Decorin |
| DOCK4 | Dedicator of Cytokinesis 4 |
| DPYSL4 | Dihydropyrimidinase Like 4 |
| EEF1A1 | Eukaryotic Translation Elongation Factor 1 Alpha 1 |
| ENAH | Enabled Homolog |
| ENC1 | Ectodermal-Neural Cortex 1 |
| EPHA7 | EPH Receptor A7 |
| ETV1 | ETS Variant Transcription Factor 1 |
| ETV5 | ETS Variant Transcription Factor 5 |
| FEZF2 | FEZ Family Zinc Finger 2 |
| FLRT1 | Fibronectin Leucine Rich Transmembrane Protein 1 |
| FLRT3 | Fibronectin Leucine Rich Transmembrane Protein 3 |
| FOS | Fos Proto-Oncogene, AP-1 Transcription Factor Subunit |
| FOXG1 | Forkhead Box G1 |
| GABRA1 | Gamma-Aminobutyric Acid Type A Receptor Subunit Alpha1 |
| GAD1 | Glutamate Decarboxylase 1 |
| GAD2 | Glutamate Decarboxylase 2 |
| GADD45G | Growth Arrest and DNA Damage Inducible Gamma |
| GFAP | Glial Fibrillary Acidic Protein |
| GNAL | G Protein Subunit Alpha L |
| GNAQ | G Protein Subunit Alpha Q |
| GRIA1 | Glutamate Ionotropic Receptor AMPA Type Subunit 1 |
| GRIA2 | Glutamate Ionotropic Receptor AMPA Type Subunit 2 |
| GRID2 | Glutamate Ionotropic Receptor Delta Type Subunit 2 |
| GRIP1 | Glutamate Receptor Interacting Protein 1 |
| GRK3 | G Protein-Coupled Receptor Kinase 3 |
| GTF2H2 | General Transcription Factor IIH Subunit 2 |
| HES6 | Hes Family BHLH Transcription Factor 6 |
| HPCA | Hippocalcin |
| ITGA7 | Integrin Subunit Alpha 7 |
| ITGA8 | Integrin Subunit Alpha 8 |
| JAG1 | Jagged Canonical Notch Ligand 1 |
| JUN | Jun Proto-Oncogene, AP-1 Transcription Factor Subunit |
| KCNIP2 | Potassium Voltage-Gated Channel Interacting Protein 2 |
| KCNJ3 | Potassium Inwardly Rectifying Channel Subfamily J Member 3 |
| KCNJ6 | Potassium Inwardly Rectifying Channel Subfamily J Member 6 |
| KLHL2 | Kelch Like Family Member 2 |
| KLHL5 | Kelch Like Family Member 5 |
| KSR1 | Kinase Suppressor of Ras 1 |
| LRRTM3 | Leucine Rich Repeat Transmembrane Neuronal 3 |
| MAG | Myelin Associated Glycoprotein |
| MAML2 | Mastermind Like Transcriptional Coactivator 2 |
| MAP4 | Microtubule Associated Protein 4 |
| MAPK1 | Mitogen-Activated Protein Kinase 1 |
| MBP | Myelin Basic Protein |
| MCOLN3 | Mucolipin TRP Cation Channel 3 |
| MEF2A | Myocyte Enhancer Factor 2A |
| MEF2C | Myocyte Enhancer Factor 2C |
| MLTT3 | Mixed-Lineage Leukemia Translocated to 3 |
| MOG | Myelin Oligodendrocyte Glycoprotein |
| MT-ND1 | Mitochondrially Encoded NADH:Ubiquinone Oxidoreductase Core Subunit 1 |
| MT-ND4L | Mitochondrially Encoded NADH:Ubiquinone Oxidoreductase Core Subunit 4L |
| MT-ND5 | Mitochondrially Encoded NADH:Ubiquinone Oxidoreductase Core Subunit 5 |
| MT2A | Metallothionein 2A |
| MT3 | Metallothionein 3 |
| MYT1L | Myelin Transcription Factor 1 Like |
| NACC2 | Nucleus Accumbens Associated 2 |
| NAPB | NSF Attachment Protein Beta |
| NCDN | Neurochondrin |
| NCOA1 | Nuclear Receptor Coactivator 1 |
| NEFM | Neurofilament Medium |
| NEUROD6 | Neuronal Differentiation 6 |
| NFIA | Nuclear Factor I A |
| NNAT | Neuronatin |
| NPAS3 | Neuronal PAS Domain Protein 3 |
| NPTXR | Neuronal Pentraxin Receptor |
| NRG3 | Neuregulin 3 |
| NRGN | Neurogranin |
| NRP1 | Neuropilin 1 |
| NRP2 | Neuropilin 2 |
| NSMF | NMDA Receptor Synaptonuclear Signaling and Neuronal Migration Factor |
| NXPH1 | Neurexophilin 1 |
| OLIG1 | Oligodendrocyte Transcription Factor 1 |
| OLIG2 | Oligodendrocyte Transcription Factor 2 |
| PALMD | Palmdelphin |
| PAX6 | Paired Box 6 |
| PCDH11X | Protocadherin 11 X-Linked |
| PCDH15 | Protocadherin Related 15 |
| PCDH8 | Protocadherin 8 |
| PCDH9 | Protocadherin 9 |
| PCSK5 | Proprotein Convertase Subtilisin/Kexin Type 5 |
| PEG10 | Paternally Expressed 10 |
| PEG3 | Paternally Expressed 3 |
| PLPPR4 | Phospholipid Phosphatase Related 4 |
| PLXNA4 | Plexin A4 |
| PRKCA | Protein Kinase C Alpha |
| PRKCE | Protein Kinase C Epsilon |
| PRKCG | Protein Kinase C Gamma |
| PRNP | Prion Protein |
| PROX1 | Prospero Homeobox 1 |
| PTK2B | Protein Tyrosine Kinase 2 Beta |
| PTPRC | Protein Tyrosine Phosphatase Receptor Type C (CD45) |
| PTPRD | Protein Tyrosine Phosphatase Receptor Type D |
| PTPRZ1 | Protein Tyrosine Phosphatase Receptor Type Z1 |
| RBFOX3 | RNA Binding Fox-1 Homolog 3 |
| RIN1 | Ras and Rab Interactor 1 |
| SATB2 | SATB Homeobox 2 |
| SEMA5A | Semaphorin 5A |
| SGK1 | Serum/Glucocorticoid Regulated Kinase 1 |
| SHISA6 | Shisa Family Member 6 |
| SLC17A7 | Solute Carrier Family 17 Member 7 (VGLUT1) |
| SLC1A2 | Solute Carrier Family 1 Member 2 |
| SLC7A11 | Solute Carrier Family 7 Member 11 |
| SLIT1 | Slit Guidance Ligand 1 |
| SLIT2 | Slit Guidance Ligand 2 |
| SLIT3 | Slit Guidance Ligand 3 |
| SNAP25 | Synaptosome Associated Protein 25 |
| SORT1 | Sortilin 1 |
| SOX11 | SRY-Box Transcription Factor 11 |
| SPARCL1 | SPARC Like 1 |
| SPON1 | Spondin 1 |
| SSTR2 | Somatostatin Receptor 2 |
| SYNGR1 | Synaptogyrin 1 |
| SYNPR | Synaptoporin |
| TANC1 | Tetratricopeptide Repeat, Ankyrin Repeat And Coiled-Coil Containing 1 |
| TBR1 | T-Box Brain Transcription Factor 1 |
| THY1 | Thy-1 Cell Surface Antigen |
| TIAM1 | TIAM Rac1 Associated GEF 1 |
| TLE4 | Transducin Like Enhancer Of Split 4 |
| TMEM215 | Transmembrane Protein 215 |
| TMSB4X | Thymosin Beta 4 X-Linked |
| TRPM3 | Transient Receptor Potential Cation Channel Subfamily M Member 3 |
| UNC5D | Unc-5 Netrin Receptor D |
| WIPF3 | WASP Interacting Protein Family Member 3 |
| ZBTB20 | Zinc Finger and BTB Domain Containing 20 |
| ZFHX4 | Zinc Finger Homeobox 4 |
| ZFPM2 | Zinc Finger Protein, FOG Family Member 2 |
| ZIC1 | Zic Family Member 1 |
| ZNF608 | Zinc Finger Protein 608 |

## Supplementary Table 5

**Supplementary Table 5 R code. Information on the script set in order to combine the snRNAseq data from developmental human brain at different stages, together with the snRNAseq data of the temporal lobe and GG samples. The snRNAseq data from human brain development have been published by PMID: 36318921. Single-nuclei RNA-seq and single-nuclei ATAC-seq data have been deposited at GEO under accession number GEO:GSE168408. Raw data from temporal lobe and GG samples are submitted under the accession number: SUB15314645**

| **Information** | **Code** |
| --- | --- |
| Install packages | install.packages("Seurat") # CRAN version  install.packages("SeuratObject")  install.packages("EnhancedVolcano")  install.packages("hdf5r")  install.packages("R.utils")  install.packages("remotes")  install.packages("devtools")  ## Load libraries  library(hdf5r)  library(patchwork)  library(stringr)  library(Seurat)  library(ggplot2)  library(tidyverse)  library(dplyr)  library(ggrepel)  library(EnhancedVolcano)  ## Version check  packageVersion("Seurat")  packageVersion("SeuratObject")  ## Bioconductor and monocle3 installation  if (!requireNamespace("BiocManager", quietly = TRUE)) install.packages("BiocManager")  BiocManager::install(version = "3.17")  BiocManager::install(c("BiocGenerics", "DelayedArray", "DelayedMatrixStats",  "limma", "lme4", "S4Vectors", "SingleCellExperiment",  "SummarizedExperiment", "batchelor", "HDF5Array",  "terra", "ggrastr"), force = TRUE)  devtools::install_github("cole-trapnell-lab/monocle3", ref = "develop", force = TRUE)  library(monocle3)  ## Install Seurat-Wrappers  remotes::install_github("satijalab/seurat-wrappers", force = TRUE)  library(SeuratWrappers) |
| Upload files and create Seurat objects | # Fetal - Gestational age: (ga22, ga24)  ga22_counts <- Read10X_h5("~/Dropbox (Uni-Klinik Bonn)/03_rstudio/geo_GSE168408/GSM5138509_RL2103_ga22_snRNAseq_filtered_feature_bc_matrix.h5", use.names = TRUE, unique.features = TRUE)  ga22 <- CreateSeuratObject(counts = ga22_counts, min.features = 100)  ga24_counts <- Read10X_h5("~/Dropbox (Uni-Klinik Bonn)/03_rstudio/geo_GSE168408/GSM5138511_RL2107_ga24_snRNAseq_filtered_feature_bc_matrix.h5", use.names = TRUE, unique.features = TRUE)  ga24 <- CreateSeuratObject(counts = ga24_counts, min.features = 100)  combined_fetal <- merge(x = ga22, y = ga24, add.cell.ids = c("ga22", "ga24"), project = "ga")  # Neonatal: (>=ga38 to <2 months)  pn1m_counts <- Read10X_h5("~/Dropbox (Uni-Klinik Bonn)/03_rstudio/geo_GSE168408/GSM5138514_RL1777_1m_snRNAseq_filtered_feature_bc_matrix.h5", use.names = TRUE, unique.features = TRUE)  pn1m <- CreateSeuratObject(counts = pn1m_counts, min.features = 100)  pn2m_counts <- Read10X_h5("~/Dropbox (Uni-Klinik Bonn)/03_rstudio/geo_GSE168408/GSM5138516_RL1612_2m_snRNAseq_filtered_feature_bc_matrix.h5", use.names = TRUE, unique.features = TRUE)  pn2m <- CreateSeuratObject(counts = pn2m_counts, min.features = 100)  pn3m_counts <- Read10X_h5("~/Dropbox (Uni-Klinik Bonn)/03_rstudio/geo_GSE168408/GSM5138517_RL2100_3m_snRNAseq_filtered_feature_bc_matrix.h5", use.names = TRUE, unique.features = TRUE)  pn3m <- CreateSeuratObject(counts = pn3m_counts, min.features = 100)  combined_neonatal <- merge(x = pn1m, y = c(pn2m, pn3m), add.cell.ids = c("pn1m", "pn2m", "pn3m"), project = "neonatal")  # Children (>=1 year to <10 years)  pn2y_b_counts <- Read10X_h5("~/Dropbox (Uni-Klinik Bonn)/03_rstudio/geo_GSE168408/GSM5138527_RL1786_2y_b_filtered_feature_bc_matrix.h5", use.names = TRUE, unique.features = TRUE)  pn2y <- CreateSeuratObject(counts = pn2y_b_counts, min.features = 100)  pn3y_counts <- Read10X_h5("~/Dropbox (Uni-Klinik Bonn)/03_rstudio/geo_GSE168408/GSM5138530_RL2129_3y_snRNAseq_filtered_feature_bc_matrix.h5", use.names = TRUE, unique.features = TRUE)  pn3y <- CreateSeuratObject(counts = pn3y_counts, min.features = 100)  pn4y_counts <- Read10X_h5("~/Dropbox (Uni-Klinik Bonn)/03_rstudio/geo_GSE168408/GSM5138531_RL2109_4y_snRNAseq_filtered_bc_matrix.h5", use.names = TRUE, unique.features = TRUE)  pn4y <- CreateSeuratObject(counts = pn4y_counts, min.features = 100)  combined_child <- merge(x = pn2y, y = c(pn3y, pn4y), add.cell.ids = c("pn2y", "pn3y", "pn4y"), project = "child")  # Adult (>=20 years)  pn20y_a_counts <- Read10X_h5("~/Dropbox (Uni-Klinik Bonn)/03_rstudio/geo_GSE168408/GSM5138546_RL2123_20y_a_snRNAseq_filtered_feature_bc_matrix.h5", use.names = TRUE, unique.features = TRUE)  pn20y <- CreateSeuratObject(counts = pn20y_a_counts, min.features = 100)  pn25y_b_counts <- Read10X_h5("~/Dropbox (Uni-Klinik Bonn)/03_rstudio/geo_GSE168408/GSM5138549_RL2132_25y_snRNAseq_filtered_feature_bc_matrix.h5", use.names = TRUE, unique.features = TRUE)  pn25y <- CreateSeuratObject(counts = pn25y_b_counts, min.features = 100)  pn17y_b_counts <- Read10X_h5("~/Dropbox (Uni-Klinik Bonn)/03_rstudio/geo_GSE168408/GSM5138545_RL2131_17y_snRNAseq_filtered_feature_bc_matrix.h5", use.names = TRUE, unique.features = TRUE)  pn17y <- CreateSeuratObject(counts = pn17y_b_counts, min.features = 100)  combined_adult <- merge(x = pn20y, y = c(pn25y, pn17y), add.cell.ids = c("pn20y", "pn25y", "pn17y"), project = "combined_adult")  # GG samples  GG1_counts <- Read10X(data.dir = "~/Dropbox (Uni-Klinik Bonn)/03_rstudio/exon9/filtered_feature_bc_matrix")  GG1 <- CreateSeuratObject(counts = GG1_counts, min.features = 100)  GG2_counts <- Read10X(data.dir = "~/Dropbox (Uni-Klinik Bonn)/03_rstudio/exon10/filtered_feature_bc_matrix")  GG2 <- CreateSeuratObject(counts = GG2_counts, min.features = 100)  GG4_counts <- Read10X(data.dir = "~/Dropbox (Uni-Klinik Bonn)/03_rstudio/exon12/filtered_feature_bc_matrix")  GG4 <- CreateSeuratObject(counts = GG4_counts, min.features = 100)  GG5_counts <- Read10X(data.dir = "~/Dropbox (Uni-Klinik Bonn)/03_rstudio/exon13/filtered_feature_bc_matrix")  GG5 <- CreateSeuratObject(counts = GG5_counts, min.features = 100)  combined_GG_adult <- merge(x = GG2, y = c(GG4, GG1, GG5), add.cell.ids = c("GG2", "GG4", "GG1", "GG5"), project = "GG_adult")  GG3_counts <- Read10X(data.dir = "~/Dropbox (Uni-Klinik Bonn)/03_rstudio/exon11/filtered_feature_bc_matrix")  GG3 <- CreateSeuratObject(counts = GG3_counts, min.features = 100, project = "GG_child")  # TLE (control) samples  TLE1_counts <- Read10X(data.dir = "~/Dropbox (Uni-Klinik Bonn)/03_rstudio/exon14/filtered_feature_bc_matrix")  TLE1 <- CreateSeuratObject(counts = TLE1_counts, min.features = 100)  TLE2_counts <- Read10X(data.dir = "~/Dropbox (Uni-Klinik Bonn)/03_rstudio/exon15/filtered_feature_bc_matrix")  TLE2 <- CreateSeuratObject(counts = TLE2_counts, min.features = 100)  TLE3_counts <- Read10X(data.dir = "~/Dropbox (Uni-Klinik Bonn)/03_rstudio/exon16/filtered_feature_bc_matrix")  TLE3 <- CreateSeuratObject(counts = TLE3_counts, min.features = 100)  combined_TLE <- merge(x = TLE1, y = c(TLE2, TLE3), add.cell.ids = c("TLE1", "TLE2", "TLE3"), project = "TLE")  # Merge all datasets into one  merged_datasets_dev <- merge(x = combined_fetal,  y = list(combined_neonatal, combined_child, combined_adult, GG3, combined_GG_adult, combined_TLE),  add.cell.ids = c("fetal", "neonatal", "child", "adult", "GG_child", "GG_adult", "TLE"),  project = "merged") |
| Integrate data | # Create metadata  metadata <- merged_datasets_dev@meta.data  metadata$cells <- rownames(metadata)  merged_datasets_dev$cells <- metadata$cells  # Annotate sample group  merged_datasets_dev$sample <- NA  merged_datasets_dev$sample[which(str_detect(merged_datasets_dev$cells, "^fetal_"))] <- "fetal"  merged_datasets_dev$sample[which(str_detect(merged_datasets_dev$cells, "^neonatal_"))] <- "neonatal"  merged_datasets_dev$sample[which(str_detect(merged_datasets_dev$cells, "^child_"))] <- "child"  merged_datasets_dev$sample[which(str_detect(merged_datasets_dev$cells, "^adult_"))] <- "adult"  merged_datasets_dev$sample[which(str_detect(merged_datasets_dev$cells, "^GG_child_"))] <- "GG_child"  merged_datasets_dev$sample[which(str_detect(merged_datasets_dev$cells, "^GG_adult_"))] <- "GG_adult"  merged_datasets_dev$sample[which(str_detect(merged_datasets_dev$cells, "^TLE_"))] <- "TLE"  # Annotate replicate  merged_datasets_dev$replicate <- NA  merged_datasets_dev$replicate[which(str_detect(metadata$cells, "^fetal_ga22_"))] <- "ga22"  merged_datasets_dev$replicate[which(str_detect(metadata$cells, "^fetal_ga24_"))] <- "ga24"  merged_datasets_dev$replicate[which(str_detect(metadata$cells, "^neonatal_pn1m_"))] <- "pn1m"  merged_datasets_dev$replicate[which(str_detect(metadata$cells, "^neonatal_pn2m_"))] <- "pn2m"  merged_datasets_dev$replicate[which(str_detect(metadata$cells, "^neonatal_pn3m_"))] <- "pn3m"  merged_datasets_dev$replicate[which(str_detect(metadata$cells, "^child_pn2y_"))] <- "pn2y"  merged_datasets_dev$replicate[which(str_detect(metadata$cells, "^child_pn3y_"))] <- "pn3y"  merged_datasets_dev$replicate[which(str_detect(metadata$cells, "^child_pn4y_"))] <- "pn4y"  merged_datasets_dev$replicate[which(str_detect(metadata$cells, "^adult_pn20y"))] <- "pn20y"  merged_datasets_dev$replicate[which(str_detect(metadata$cells, "^adult_pn25y"))] <- "pn25y"  merged_datasets_dev$replicate[which(str_detect(metadata$cells, "^adult_pn17y"))] <- "pn17y"  merged_datasets_dev$replicate[which(str_detect(metadata$cells, "^GG_adult_GG1_"))] <- "GG1_adult"  merged_datasets_dev$replicate[which(str_detect(metadata$cells, "^GG_adult_GG2_"))] <- "GG2_adult"  merged_datasets_dev$replicate[which(str_detect(metadata$cells, "^GG_child_GG3_"))] <- "GG3_child"  merged_datasets_dev$replicate[which(str_detect(metadata$cells, "^GG_adult_GG4_"))] <- "GG4_adult"  merged_datasets_dev$replicate[which(str_detect(metadata$cells, "^GG_adult_GG5_"))] <- "GG5_adult"  merged_datasets_dev$replicate[which(str_detect(metadata$cells, "^TLE_TLE1_"))] <- "TLE1"  merged_datasets_dev$replicate[which(str_detect(metadata$cells, "^TLE_TLE2_"))] <- "TLE2"  merged_datasets_dev$replicate[which(str_detect(metadata$cells, "^TLE_TLE3_"))] <- "TLE3"  # Annotate dataset origin  merged_datasets_dev$dataset <- NA  merged_datasets_dev$dataset[which(str_detect(merged_datasets_dev$cells, "^fetal_"))] <- "dataset2"  merged_datasets_dev$dataset[which(str_detect(merged_datasets_dev$cells, "^neonatal_"))] <- "dataset2"  merged_datasets_dev$dataset[which(str_detect(merged_datasets_dev$cells, "^child_"))] <- "dataset2"  merged_datasets_dev$dataset[which(str_detect(merged_datasets_dev$cells, "^adult_"))] <- "dataset2"  merged_datasets_dev$dataset[which(str_detect(merged_datasets_dev$cells, "^GG_child_"))] <- "dataset1"  merged_datasets_dev$dataset[which(str_detect(merged_datasets_dev$cells, "^GG_adult_"))] <- "dataset1"  merged_datasets_dev$dataset[which(str_detect(merged_datasets_dev$cells, "^TLE_"))] <- "dataset1"  # QC metrics  merged_datasets_dev$log10GenesPerUMI <- log10(merged_datasets_dev$nFeature_RNA) / log10(merged_datasets_dev$nCount_RNA)  merged_datasets_dev$mitoRatio <- PercentageFeatureSet(merged_datasets_dev, pattern = "^MT-") / 100  merged_datasets_dev$percent.Ribosomal <- PercentageFeatureSet(merged_datasets_dev, pattern = "^RP[LS]")  # Cell filtering  filtered_dev <- subset(merged_datasets_dev,  subset = (nCount_RNA >= 500) &  (nFeature_RNA > 250) &  (log10GenesPerUMI > 0.80) &  (mitoRatio < 0.2))  # Gene filtering (≥10 cells)  counts <- GetAssayData(filtered_dev, slot = "counts")  keep_genes <- Matrix::rowSums(counts > 0) >= 10  filtered_counts <- counts[keep_genes, ]  filtered_dev <- CreateSeuratObject(filtered_counts, meta.data = filtered_dev@meta.data)  metadata <- filtered_dev@meta.data  # Cell count summary  table(filtered_dev$sample)  # RPCA Integration  gg.list <- SplitObject(filtered_dev, split.by = "dataset")  gg.list <- lapply(gg.list, function(x) {  x <- NormalizeData(x, verbose = FALSE)  x <- FindVariableFeatures(x, verbose = FALSE, nfeatures = 2000)  })  features <- SelectIntegrationFeatures(object.list = gg.list)  gg.list <- lapply(gg.list, function(x) {  x <- ScaleData(x, features = features, verbose = FALSE)  x <- RunPCA(x, features = features, verbose = FALSE)  })  anchors <- FindIntegrationAnchors(object.list = gg.list, reduction = "rpca", dims = 1:10)  integrated_dev_02 <- IntegrateData(anchorset = anchors, dims = 1:10)  # Post-integration workflow  integrated_dev_02 <- FindVariableFeatures(integrated_dev_02, selection.method = "vst", nfeatures = 2000)  integrated_dev_02 <- ScaleData(integrated_dev_02, verbose = FALSE)  integrated_dev_02 <- RunPCA(integrated_dev_02, npcs = 50, verbose = FALSE)  integrated_dev_02 <- RunUMAP(integrated_dev_02, reduction = "pca", dims = 1:10)  integrated_dev_02 <- FindNeighbors(integrated_dev_02, reduction = "pca", dims = 1:10)  integrated_dev_02 <- FindClusters(integrated_dev_02, resolution = 0.5)  # Backup cluster identities  integrated_dev_02$old.idents <- Idents(integrated_dev_02)  # Cell count summary post-integration  table(integrated_dev_02$sample)  table(integrated_dev_02$replicate)  # Marker discovery  DefaultAssay(integrated_dev_02) <- "RNA"  Idents(integrated_dev_02) <- integrated_dev_02$seurat_clusters  markers <- FindAllMarkers(integrated_dev_02, only.pos = TRUE, logfc.threshold = 0.25)  # Save object  save(integrated_dev_02, file = "~/Dropbox (Uni-Klinik Bonn)/03_rstudio/+datasets/02_developmental/integrated_dev_02_old.idents.RData") |
| Expression levels of cell-specific markers | # According to cell markers (GG paper)  DefaultAssay(integrated_dev_02) <- "RNA"  Idents(integrated_dev_02) <- integrated_dev_02$seurat_clusters  # Neuron annotation  ## Immature principal neurons  VlnPlot(integrated_dev_02, features = c("DCX"), pt.size = 0, combine = FALSE, raster = FALSE)  FeaturePlot(integrated_dev_02, features = c("DCX"), raster = FALSE)  ## General neuronal markers  VlnPlot(integrated_dev_02, features = c("RBFOX3"), pt.size = 0, combine = FALSE, raster = FALSE)  FeaturePlot(integrated_dev_02, features = c("RBFOX3"), raster = FALSE)  VlnPlot(integrated_dev_02, features = c("SNAP25"), pt.size = 0, combine = FALSE)  FeaturePlot(integrated_dev_02, features = c("SNAP25"), raster = FALSE)  VlnPlot(integrated_dev_02, features = c("MAP2"), pt.size = 0, combine = FALSE)  FeaturePlot(integrated_dev_02, features = c("MAP2"), raster = FALSE)  ## Principal neurons L2-3 (Immature)  VlnPlot(integrated_dev_02, features = c("SATB2"), pt.size = 0, combine = FALSE, raster = FALSE)  FeaturePlot(integrated_dev_02, features = c("SATB2"), raster = FALSE)  VlnPlot(integrated_dev_02, features = c("FAM19A2", "FGF13", "MEF2C", "STMN2"), pt.size = 0, combine = FALSE, raster = FALSE)  lapply(c("FAM19A2", "FGF13", "MEF2C", "STMN2"), function(g) FeaturePlot(integrated_dev_02, features = g, raster = FALSE))  ## Principal neurons L2-3 (Mature)  VlnPlot(integrated_dev_02, features = c("CAMK2A"), pt.size = 0, combine = FALSE, raster = FALSE)  FeaturePlot(integrated_dev_02, features = c("CAMK2A"), raster = FALSE)  ## Principal neurons L4 (Immature)  VlnPlot(integrated_dev_02, features = c("RORB"), pt.size = 0, combine = FALSE, raster = FALSE)  FeaturePlot(integrated_dev_02, features = c("RORB"), raster = FALSE)  ## Excitatory neurons  exc_genes <- c("CUX2", "NRGN", "SLC17A7")  lapply(exc_genes, function(g) {  VlnPlot(integrated_dev_02, features = g, pt.size = 0, combine = FALSE, raster = FALSE)  FeaturePlot(integrated_dev_02, features = g, raster = FALSE)  })  ## Inhibitory neurons  inh_genes <- c("DLX1", "DLX2", "LHX6", "GAD1", "SLC32A1", "SST", "VIP")  lapply(inh_genes, function(g) {  VlnPlot(integrated_dev_02, features = g, pt.size = 0, combine = FALSE, raster = FALSE)  FeaturePlot(integrated_dev_02, features = g, raster = FALSE)  })  # Other inhibitory neuron markers  other_inh <- c("RELN", "SV2C", "KIT", "CXCL14", "LAMP5", "CCK", "CA1", "CALB2")  lapply(other_inh, function(g) FeaturePlot(integrated_dev_02, features = g, raster = FALSE))  FeaturePlot(integrated_dev_02, features = c("CD34"), raster = FALSE, order = TRUE, pt.size = 0)  # Astrocytes  astro_genes <- c("GFAP", "AQP4", "ALDH1L1", "SLC1A3", "SLC1A2")  lapply(astro_genes, function(g) {  VlnPlot(integrated_dev_02, features = g, pt.size = 0, combine = FALSE, raster = FALSE, group.by = "celltype")  FeaturePlot(integrated_dev_02, features = g, raster = FALSE)  })  # Oligodendrocytes  oligo_genes <- c("MBP", "MOG", "CNP", "MAG", "PLP1")  lapply(oligo_genes, function(g) {  VlnPlot(integrated_dev_02, features = g, pt.size = 0, combine = FALSE, raster = FALSE, group.by = "celltype")  FeaturePlot(integrated_dev_02, features = g, raster = FALSE)  })  # OPCs  opc_genes <- c("GPR17", "CSPG4", "OLIG1", "OLIG2", "PDGFRA")  lapply(opc_genes, function(g) {  VlnPlot(integrated_dev_02, features = g, pt.size = 0, combine = FALSE, raster = FALSE)  FeaturePlot(integrated_dev_02, features = g, raster = FALSE)  })  # Microglia  microglia_genes <- c("PTPRC", "CD14", "P2RY12", "C1QA")  lapply(microglia_genes, function(g) {  VlnPlot(integrated_dev_02, features = g, pt.size = 0, raster = FALSE)  FeaturePlot(integrated_dev_02, features = g, raster = FALSE)  })  # Endothelial  endothelial_genes <- c("VWF", "PECAM1", "CD34")  lapply(endothelial_genes, function(g) {  VlnPlot(integrated_dev_02, features = g, pt.size = 0, combine = FALSE, raster = FALSE)  FeaturePlot(integrated_dev_02, features = g, raster = FALSE)  })  # Vasculature  vasculature_genes <- c("CLDN5", "TBX18")  lapply(vasculature_genes, function(g) {  VlnPlot(integrated_dev_02, features = g, pt.size = 0, raster = FALSE)  FeaturePlot(integrated_dev_02, features = g, raster = FALSE)  })  # VLMCs  vlmc_genes <- c("DCN", "COL1A1", "COL1A2")  lapply(vlmc_genes, function(g) VlnPlot(integrated_dev_02, features = g, pt.size = 0, combine = FALSE, raster = FALSE)) |
| Rename clusters | #Add new name to the clusters and save  new.cluster.ids <- c("Oligodendroglia", "NPCs", "Ex_neurons", "In_neurons","Astroglia", "Astroglia", "OPCs", "Ex_neurons", "Astroglia", "Ex_neurons", "Astroglia", "Microglia", "NPCs" ,"NPCs", "Ex_neurons", "Ex_neurons", "In_neurons", "Ex_neurons", "Ex_neurons", "NPCs" , "Oligodendroglia", "Endothelial", "Oligodendroglia", "Astroglia", "Oligodendroglia", "Ex_neurons", "Ex_neurons", "OPCs")  names(new.cluster.ids) <- levels(integrated_dev_02)  integrated_dev_02 <- RenameIdents(integrated_dev_02, new.cluster.ids)  #Add a new column in metadata for the cell type  integrated_dev_02$celltype <- Idents(integrated_dev_02)  #How many cells per cell type  table(integrated_dev_02$celltype)  #How many cells per cell type and condition?  table(integrated_dev_02@meta.data$celltype , integrated_dev_02@meta.data$sample)  #Show the Dimplot  #(07_#DimPlot_celltype)  DimPlot(integrated_dev_02 , reduction = "umap", group.by="celltype",  cols = c('NPCs'='#5f6f52','Ex_neurons'='#a9b388','In_neurons'='#D4D2A5','Oligodendroglia'="#E7C1A5" ,'OPCs'='#c4661f', 'Astroglia'='#d9b0b1','Microglia'='#783d19',  'Endothelial'='#96C0B7'), raster = FALSE)  #(07_#DimPlot_celltype_new_colors)  DimPlot(integrated_dev_02 , reduction = "umap", group.by="celltype",  cols = c('NPCs'='#4477AA','Ex_neurons'='#EE6677','In_neurons'='#228833','Oligodendroglia'="#CCBB44" ,'OPCs'='#E7C1A5', 'Astroglia'='#AA3377','Microglia'='#E17C05',  'Endothelial'='#000000'), raster = FALSE)  #(08_#Dimplot_celltype@replicate)  DimPlot(integrated_dev_02, reduction = "umap", group.by="celltype", label = FALSE, split.by = "replicate",  cols = c('NPCs'='#5f6f52','Ex_neurons'='#a9b388','In_neurons'='#D4D2A5','Oligodendroglia'="#E7C1A5" ,'OPCs'='#c4661f', 'Astroglia'='#d9b0b1','Microglia'='#783d19',  'Endothelial'='#96C0B7'), raster = FALSE)  #Visualize cluster proportion  #basic plot of clusters by replicate (09_#StackedPlot_Clusters@sample)  ggplot(integrated_dev_02@meta.data, aes(x=celltype, fill= factor(sample, levels = c("fetal", "neonatal", "child", "infancy", "adult", "GG_adult", "GG_child", "TLE")))) + geom_bar() +  scale_fill_manual(values = c('fetal'='#440154', 'neonatal'='#31688e',"child" = "#35b779", 'adult'='#fde725', 'TLE'='#00AEEF','GG_child'='#FFBEB2',"GG_adult" = "red"))+  xlab("Clusters") + ylab("Frequency")+  theme_classic()  #plot as proportion or percentage of cluster (10_#StackedPlot_Percentage_clusters@sample)  ggplot(integrated_dev_02@meta.data, aes(x=celltype, fill= factor(sample, levels = c("fetal", "neonatal", "child", "infancy", "adult", "GG_adult", "GG_child", "TLE")))) + geom_bar(position = "fill")+  scale_fill_manual(values = c('fetal'='#440154', 'neonatal'='#31688e',"child" = "#35b779", 'adult'='#fde725', 'TLE'='#00AEEF','GG_child'='#FFBEB2',"GG_adult" = "red"))+  xlab("Clusters") + ylab("Frequency")+  theme_classic()  #basic plot of cell types for each sample of origin (11_#StackedPlot_celltype@sample)  ggplot(integrated_dev_02@meta.data, aes(x=sample, fill= factor(celltype, levels = c("Ex_neurons", "In_neurons", "OPCs", "Microglia", "NPCs", "Oligodendroglia", "Astroglia", "Endothelial")))) + geom_bar( color = "black") +  scale_fill_manual(values = c('NPCs'='#5f6f52','Ex_neurons'='#a9b388','In_neurons'='#D4D2A5','Oligodendroglia'="#E7C1A5" ,'OPCs'='#c4661f', 'Astroglia'='#d9b0b1','Microglia'='#783d19',  'Endothelial'='#96C0B7'))+  scale_x_discrete(limit = c('fetal', 'neonatal',"child" , 'adult', 'TLE','GG_child',"GG_adult" ))+  xlab("Cell Types") + ylab("Frequency")+  theme_classic()  #basic plot of cell types for each sample of origin (11_#StackedPlot_celltype@sample_new_colors)  ggplot(integrated_dev_02@meta.data, aes(x=sample, fill= factor(celltype, levels = c("Ex_neurons", "In_neurons", "OPCs", "Microglia", "NPCs", "Oligodendroglia", "Astroglia", "Endothelial")))) + geom_bar( color = "black") +  scale_fill_manual(values = c('NPCs'='#4477AA','Ex_neurons'='#EE6677','In_neurons'='#228833','Oligodendroglia'="#CCBB44" ,'OPCs'='#E7C1A5', 'Astroglia'='#AA3377','Microglia'='#E17C05',  'Endothelial'='#000000'))+  scale_x_discrete(limit = c('fetal', 'neonatal',"child" , 'adult', 'TLE','GG_child',"GG_adult" ))+  xlab("Cell Types") + ylab("Frequency")+  theme_classic()  #plot as proportion or percentage of cell types (12_#StackedPlot_Percentage_celltype@sample)  ggplot(integrated_dev_02@meta.data, aes(x=sample, fill= factor(celltype, levels = c("NPCs", "Ex_neurons", "In_neurons", "Oligodendroglia", "OPCs", "Astroglia", "Microglia" ,"Endothelial")))) + geom_bar(position = "fill", color = "black")+  scale_fill_manual(values = c('NPCs'='#5f6f52','Ex_neurons'='#a9b388','In_neurons'='#D4D2A5','Oligodendroglia'="#E7C1A5" ,'OPCs'='#c4661f', 'Astroglia'='#d9b0b1','Microglia'='#783d19',  'Endothelial'='#96C0B7'))+  scale_x_discrete(limit = c('fetal', 'neonatal',"child" , 'adult', 'TLE','GG_child',"GG_adult" ))+  xlab("Clusters") + ylab("Frequency")+  theme_classic()  #plot as proportion or percentage of cell types (12_#StackedPlot_Percentage_celltype@sample_new_colors)  ggplot(integrated_dev_02@meta.data, aes(x=sample, fill= factor(celltype, levels = c("NPCs", "Ex_neurons", "In_neurons", "Oligodendroglia", "OPCs", "Astroglia", "Microglia" ,"Endothelial")))) + geom_bar(position = "fill", color = "black")+  scale_fill_manual(values = c('NPCs'='#4477AA','Ex_neurons'='#EE6677','In_neurons'='#228833','Oligodendroglia'="#CCBB44" ,'OPCs'='#E7C1A5', 'Astroglia'='#AA3377','Microglia'='#E17C05',  'Endothelial'='#000000'))+  scale_x_discrete(limit = c('fetal', 'neonatal',"child" , 'adult', 'TLE','GG_child',"GG_adult" ))+  xlab("Clusters") + ylab("Frequency")+  theme_classic()  #Make stacked violin plot of the most common canonical markers  #Make sure that the default assay is RNA  DefaultAssay(integrated_dev_02) <- "RNA"  Idents(integrated_dev_02) <- integrated_dev_02$celltype  #Select the canonical markers  features <- c("MBP", "MOG", "MAG", "DCX", "CUX2", "SATB2", "RBFOX3", "CAMK2A", "SNAP25","SLC17A7", "NRGN", "GAD1", "GAD2",  "AQP4", "GFAP", "ALDH1L1", "OLIG1", "GPR17", "PDGFRA", "P2RY12", "PTPRC", "CLDN5", "DCN", "COL1A2")  celltype_colors <- c(  'NPCs' = '#4477AA',  'Ex_neurons' = '#EE6677',  'In_neurons' = '#228833',  'Oligodendroglia' = '#CCBB44',  'OPCs' = '#E7C1A5', # updated pastel yellow  'Astroglia' = '#AA3377',  'Microglia' = '#E17C05',  'Endothelial' = '#000000'  )  #Stacked violin plot - 13_#Stacked_violin_plot_canonical_markers  VlnPlot(  integrated_dev_02,  features = features,  stack = TRUE,  split.by = "celltype"  ) +  scale_fill_manual(values = celltype_colors) +  theme(legend.position = "none") +  ggtitle("Expression level - canonical markers")  #We find the markers for all the clusters (associated to cell type)  Idents(integrated_dev_02) <- integrated_dev_02$celltype  all_markers <- FindAllMarkers(object = integrated_dev_02,  only.pos = TRUE,  logfc.threshold = 0.25)  #Save R Data (with cell types)  save(integrated_dev_02, file="~/Dropbox (Uni-Klinik Bonn)/03_rstudio/+datasets/02_developmental/integrated_dev_02_celltype.RData")  saveRDS(integrated_dev_02, file = "~/Dropbox (Uni-Klinik Bonn)/03_rstudio/+datasets/02_developmental/integrated_dev_02_celltype.rds")  ##To avoid re-run save, I have added a random plot in this line!  ggplot(integrated_dev_02@meta.data, aes(x=sample, fill= factor(celltype, levels = c("NPCs", "Ex_neurons", "In_neurons", "Oligodendroglia", "OPCs", "Astroglia", "Microglia" ,"Endothelial")))) + geom_bar(position = "fill")+  theme_classic() |
| DEG analysis per cluster | #How many cells per cell type  table(integrated_dev_02$celltype)  #How many cells per cell type and condition?  table(integrated_dev_02@meta.data$celltype , integrated_dev_02@meta.data$sample)  #Get only 320 cells per condition and cell type  #Subset TLE and GG_adult  Idents(integrated_dev_02) <- integrated_dev_02$sample  TLE_subset <- subset(integrated_dev_02, idents = "TLE")  GG_adult_subset <- subset(integrated_dev_02, idents = "GG_adult")  #Subset cell types for TLE and GG_adult  Idents(TLE_subset) <- TLE_subset$celltype  Ex_neurons_TLE <- subset(TLE_subset, idents = "Ex_neurons", downsample=320)  Oligodendroglia_TLE <- subset(TLE_subset, idents = "Oligodendroglia", downsample=320)  In_neurons_TLE <- subset(TLE_subset, idents="In_neurons", downsample=320)  Astroglia_TLE <- subset(TLE_subset, idents="Astroglia", downsample=320)  OPCs_TLE <- subset(TLE_subset, idents = "OPCs", downsample=320)  Microglia_TLE <- subset(TLE_subset, idents = "Microglia", downsample=320)  Endothelial_TLE <- subset(TLE_subset, idents = "Endothelial", downsample=320)  Idents(GG_adult_subset) <- GG_adult_subset$celltype  Ex_neurons_GG_adult <- subset(GG_adult_subset, idents = "Ex_neurons", downsample=320)  Oligodendroglia_GG_adult <- subset(GG_adult_subset, idents = "Oligodendroglia", downsample=320)  In_neurons_GG_adult <- subset(GG_adult_subset, idents="In_neurons", downsample=320)  Astroglia_GG_adult <- subset(GG_adult_subset, idents="Astroglia", downsample=320)  OPCs_GG_adult <- subset(GG_adult_subset, idents = "OPCs", downsample=320)  Microglia_GG_adult <- subset(GG_adult_subset, idents = "Microglia", downsample=320)  Endothelial_GG_adult <- subset(GG_adult_subset, idents = "Endothelial", downsample=320)  #Check one of the cell types  table(Ex_neurons_TLE$celltype)  table(Ex_neurons_GG_adult$celltype)  #merge TLE and GG downsampled  TLE_GG_adult_merged <- merge(x=GG_adult_subset, y=TLE_subset,  add.cell.ids = c("GG_adult_subset", "TLE_subset"),  project = "combined_downsample")  TLE_GG_adult_merged_Ex_neurons <- subset(TLE_GG_adult_merged, idents = "Ex_neurons")  table(TLE_GG_adult_merged_Ex_neurons$celltype)  Ex_neurons_TLE_GG <- merge(x=Ex_neurons_TLE, y=Ex_neurons_GG_adult,  add.cell.ids = c("Ex_neurons_TLE", "Ex_neurons_GG"),  project = "Ex_neurons_merge_downsample")  table(Ex_neurons_TLE_GG$sample)  #deg GG_adult vs TLE  #Activate "sample" ident in order to be able to compare GG_adult vs adult.  Idents(Ex_neurons_TLE_GG) <- Ex_neurons_TLE_GG$sample  DefaultAssay(Ex_neurons_TLE_GG) <- "RNA"  Ex_neurons_TLE_GG <- NormalizeData(Ex_neurons_TLE_GG, verbose = FALSE)  Ex_neurons_TLE_GG_deg <- FindMarkers(Ex_neurons_TLE_GG, ident.1= "GG_adult", ident.2 = "TLE", logfc.threshold = 0.5, min.diff.pct = 0.2)  Ex_neurons_TLE_GG_deg <- Ex_neurons_TLE_GG_deg %>%  rownames_to_column(var = "GeneID")  Ex_neurons_TLE_GG_deg <- subset(Ex_neurons_TLE_GG_deg, p_val_adj < 0.05)  setwd("/Users/silvia/Dropbox (Uni-Klinik Bonn)/03_rstudio/+datasets/02_developmental/deg_normalize_cells")  write.csv(Ex_neurons_TLE_GG_deg, "Ex_neurons_GG_adult_TLE_deg.csv") |
|  |  |
| Trajectory analysis - Monocle | Idents(integrated_dev_02) <- integrated_dev_02$celltype  Ex.neuron <- subset(integrated_dev_02, idents = "Ex_neurons")  # Identify Highly Variable Features  Ex.neuron <- FindVariableFeatures(Ex.neuron, selection.method = "vst", nfeatures = 2000)  # Scale the Data  Ex.neuron <- ScaleData(Ex.neuron, verbose = FALSE)  # Run PCA  Ex.neuron <- RunPCA(Ex.neuron, npcs = 50, verbose = FALSE)  # UMAP and Clustering  Ex.neuron <- RunUMAP(Ex.neuron, reduction = "pca", dims = 1:10)  Ex.neuron <- FindNeighbors(Ex.neuron, reduction = "pca", dims = 1:10)  Ex.neuron <- FindClusters(Ex.neuron, resolution = 0.3)  # Find markers for each sample (not clusters)  markers_neurons <- FindAllMarkers(object = Ex.neuron, only.pos = TRUE, logfc.threshold = 0.25)  # DimPlot by Sample  DimPlot(Ex.neuron, reduction = "umap", group.by = "sample",  cols = c("neonatal" = '#31688e', "child" = "#35b779",  "adult" = '#fde725', "TLE" = 'lightblue',  "GG_child" = '#FFBEB2', "GG_adult" = "red"))  # DimPlot by Seurat Clusters  DimPlot(Ex.neuron, reduction = "umap", group.by = "seurat_clusters")  # Remove Fetal Samples  Idents(Ex.neuron) <- "sample"  Ex.neuron <- subset(Ex.neuron, idents = "fetal", invert = TRUE)  # Save Processed Object  save(Ex.neuron, file = "~/Dropbox (Uni-Klinik Bonn)/03_rstudio/+datasets/02_developmental/ex_neurons/pseudotime/Ex_neurons_clustered.RData")  # Save UMAP by Sample  DimPlot(Ex.neuron, group.by = "sample", label = FALSE,  cols = c("neonatal" = '#31688e', "child" = "#35b779",  "adult" = '#fde725', "TLE" = '#00AEEF',  "GG_child" = '#FFBEB2', "GG_adult" = "red"))  # Save UMAP by Clusters  DimPlot(Ex.neuron, group.by = "seurat_clusters", label = TRUE)  # Explore Metadata  head(Ex.neuron@meta.data)  # Run UMAP (extended dims)  Ex.neuron <- RunUMAP(Ex.neuron, dims = 1:20)  # Convert Seurat to Monocle3 CellDataSet  cds <- as.cell_data_set(Ex.neuron)  # Gene Metadata  fData(cds)$gene_short_name <- rownames(fData(cds))  head(fData(cds))  # View Cell Metadata and Counts  head(colData(cds))  head(counts(cds))  # Assign Uniform Partition  recreate.partitions <- as.factor(rep(1, length(cds@colData@rownames)))  names(recreate.partitions) <- cds@colData@rownames  cds@clusters@listData[["UMAP"]][["partitions"]] <- recreate.partitions  # Transfer Cluster Labels  list.cluster <- Ex.neuron@active.ident  cds@clusters@listData[["UMAP"]][["clusters"]] <- list.cluster  # Transfer UMAP Embeddings  cds@int_colData@listData[["reducedDims"]]@listData[["UMAP"]] <-  Ex.neuron@reductions$umap@cell.embeddings  # Plot Clusters Before Trajectory  cluster.before.traj <- plot_cells(cds, color_cells_by = "cluster",  label_groups_by_cluster = FALSE,  group_label_size = 5) +  theme(legend.position = "right")  cluster.before.traj  # Learn Trajectory Graph  cds <- learn_graph(cds, use_partition = FALSE)  # Plot Graph  plot_cells(cds, color_cells_by = "cluster",  label_groups_by_cluster = FALSE,  label_branch_points = TRUE,  label_roots = TRUE,  label_leaves = FALSE,  group_label_size = 5)  # Order Cells in Pseudotime  cds <- order_cells(cds, reduction_method = "UMAP", root_cells = NULL)  # Plot Pseudotime  plot_cells(cds, color_cells_by = "pseudotime",  label_groups_by_cluster = TRUE,  label_branch_points = TRUE,  label_roots = FALSE,  label_leaves = FALSE)  # Save Monocle3 Object  save(cds, file = "~/Dropbox (Uni-Klinik Bonn)/03_rstudio/+datasets/02_developmental/ex_neurons/pseudotime/csd_ex_neurons.RData")  # Extract and Save Pseudotime  cds$monocle3_pseudotime <- pseudotime(cds)  data.pseudo <- as.data.frame(colData(cds))  # Pseudotime vs Sample Boxplot  ggplot(data.pseudo, aes(monocle3_pseudotime, sample, fill = sample)) +  geom_boxplot()  # Enhanced Boxplot with Sample Ordering  ggplot(data.pseudo, aes(monocle3_pseudotime, reorder(sample, monocle3_pseudotime), fill = sample)) +  geom_boxplot() +  scale_fill_manual(values = c("#fde725", "#35b779", "red", "#FFBEB2", "#31688e", "#00AEEF")) +  theme_classic()  # Save Pseudotime Data  save(data.pseudo, file = "~/Dropbox (Uni-Klinik Bonn)/03_rstudio/+datasets/02_developmental/ex_neurons/pseudotime/data.pseudo_ex_neurons.RData") |
|  |  |
| Cell type dependent CNV analysis | # Use RNA assay to get raw counts  DefaultAssay(integrated_dev_02) <- "RNA"  counts_matrix <- as.matrix(GetAssayData(integrated_dev_02, slot = "counts"))  # Extract metadata  meta <- integrated_dev_02@meta.data  meta$cell_id <- rownames(meta)  # Subset metadata: GG_adult Ex_neurons and Endothelial cells  gg_meta <- meta[  meta$sample == "GG_adult" &  meta$celltype %in% c("Ex_neurons", "Endothelial") &  meta$cell_id %in% colnames(counts_matrix),  ]  # Check cell counts per type  table(gg_meta$celltype)  # Subset count matrix  gg_counts <- counts_matrix[, gg_meta$cell_id]  # Create annotation file: Endothelial = reference, Ex_neurons = test  cell_annotation <- data.frame(  Cell = gg_meta$cell_id,  Group = gg_meta$celltype  )  write.table(cell_annotation, "gg_ex_vs_endothelial_annotations.txt",  sep = "\t", row.names = FALSE, col.names = FALSE, quote = FALSE)  # Create inferCNV object  infercnv_obj <- CreateInfercnvObject(  raw_counts_matrix = gg_counts,  annotations_file = "gg_ex_vs_endothelial_annotations.txt",  delim = "\t",  gene_order_file = "gene_pos.txt", # Provide full path if needed  ref_group_names = c("Endothelial")  )  # Run inferCNV  infercnv_obj <- infercnv::run(  infercnv_obj,  cutoff = 0.1,  out_dir = "infercnv_GG_ex_neurons_vs_endothelial",  cluster_by_groups = TRUE,  denoise = TRUE,  HMM = TRUE,  analysis_mode = "samples"  )  # Assess CNVs in Ex_neurons, Astroglia, and Oligodendroglia in GG_adult  # Reference cells: Endothelial  # Use RNA assay to get raw counts  DefaultAssay(integrated_dev_02) <- "RNA"  counts_matrix <- as.matrix(GetAssayData(integrated_dev_02, slot = "counts"))  # Extract metadata  meta <- integrated_dev_02@meta.data  meta$cell_id <- rownames(meta)  # Subset metadata: GG_adult Astroglia, Oligodendroglia, and Endothelial  gg_meta <- meta[  meta$sample == "GG_adult" &  meta$celltype %in% c("Astroglia", "Oligodendroglia", "Endothelial") &  meta$cell_id %in% colnames(counts_matrix),  ]  # Check cell counts per type  table(gg_meta$celltype)  # Subset count matrix  gg_counts <- counts_matrix[, gg_meta$cell_id]  # Create annotation file  cell_annotation <- data.frame(  Cell = gg_meta$cell_id,  Group = gg_meta$celltype  )  write.table(cell_annotation, "gg_astro_oligo_vs_endothelial_annotations.txt",  sep = "\t", row.names = FALSE, col.names = FALSE, quote = FALSE)  # Create inferCNV object  infercnv_obj <- CreateInfercnvObject(  raw_counts_matrix = gg_counts,  annotations_file = "gg_astro_oligo_vs_endothelial_annotations.txt",  delim = "\t",  gene_order_file = "gene_pos.txt", # Provide full path if needed  ref_group_names = c("Endothelial")  )  # Run inferCNV  infercnv_obj <- infercnv::run(  infercnv_obj,  cutoff = 0.1,  out_dir = "infercnv_GG_astro_oligo_vs_endothelial",  cluster_by_groups = TRUE,  denoise = TRUE,  HMM = TRUE,  analysis_mode = "samples",  HMM_type = "i6"  ) |
